# Supplementary material for: Osteology of a forelimb of an aetosaur Stagonolepis olenkae (Archosauria: Pseudosuchia: Aetosauria) from the Krasiejów locality in Poland and its probable adaptations for a scratch-digging behavior
Source: PeerJ. 2018 Oct 2;6:e5595. doi: 10.7717/peerj.5595 (PMC6173166; doi:10.7717/peerj.5595)
Supplement: Appendix S1 [file peerj-06-5595-s001.pdf]

Appendix online 1. List of forelimb specimens of *Stagonolepis olenkae* with the description

- **ZPAL AbIII/2407** (manuscript Figure 2, supplementary Figure 1) – Specimen contains elements of the right forelimb: the ulna; the radius; three carpal elements: fused radiale and intermedium, ulnare, one distal carpal; ten elements of the manus: all metacarpals and all phalanges of the 1st row and numerous small osteoderms. The ulna and radius, are preserved in entirety and are located parallel to each other, almost as in life position. The carpus and manus are rotated at 90º in respect to the forearm bones. Most of the osteoderms are located on the dorsal side of the manus. The specimens includes also a separated cluster of osteoderms previously arranged in front of the distal end of radius and ulna (not illustrated).

- **ZPAL AbIII/3349/1** (manuscript Figure 6, supplementary Figure 2) – Specimen contains elements of the right forelimb: distal end of the ulna; distal end of the radius; four carpal elements: fused radiale and intermedium, ulnare, two distal carpals (probably the distal carpals III - smaller and IV - larger); sixteen elements of the manus: all metacarpals, all phalanges of the digit I (1st row phalanx and ungual), all phalanges of the digit II (two phalanges and ungual), broken 1st row phalanx of the digit III, four phalanges of the fourth digit IV (1st, 2nd, 3rd and 4th row phalanges), 1st row phalanx of the fifth digit; and numerous small osteoderms. The ulna and radius are preserved close to each other. The manus is rotated at 90º in respect to ulna, similar to specimen ZPAL AbIII/2407. The carpus elements are disarticulated. The fused radiale and intermedium is rotated in respect to its anatomical position (the surface visible on the dorsal side of specimen in natural arrangement should face towards the metacarpals, Fig. 2A). Rotation of this bone must have occurred when the skin still covered the limbs, as indicated by the presence of osteoderms over its surface for metacarpals. Hand elements are preserved in articulation. The metacarpal of the III digit is pathologically enlarged and thicker in respect to other metacarpals of this individual, and in comparison with other specimens with hands preserved in association (Fig. 2B). Most of the osteoderms are preserved on the dorsal side of the manus and in the sediment associated with remains of the ulna and radius. The specimen was found in assemblage with several other elements of aetosaur skeleton (collective number ZPAL AbIII/3349). It is possible that ZPAL AbIII/3349/1 and 3349/2 belong to a single animal.

- **ZPAL AbIII/3349/2** (supplementary Figure 3) – Specimen contains elements of the left forelimb: distal end of the ulna; distal end with part of the shaft of the radius; three carpal elements: fused radiale and intermedium and two other carpals (they cannot be identified properly due to poor state of preservation); ten elements of the manus: all metacarpals and five phalanges (probably 1st row phalanx of the digit III, 1st and 2nd row phalanges of the digits IV and V); numerous small osteoderms. All elements are disarticulated. It appears that at the moment of burial, the joints of the hand were pushed towards the forelimbs, which eventually caused disarticulation of the digits and displacement of its elements. On the lateral side of the fused radiale and intermedium is an unnatural hole with the rugose surface. This deformation could be caused by the decomposition process before the burial. The phalanges are in chaotic disorder and because of that it is difficult to recognize them. Most of the osteoderms are located in the sediment between the long bones and manus and in the sediment associated with the long bones (over the dorsal side of the metacarpals). The specimen was found in assemblage with several other elements of aetosaur skeleton (collective number ZPAL AbIII/3349). It is possible that ZPAL AbIII/3349/2 and 3349/1 belong to a single animal.
- **ZPAL AbIII/2071** (manuscript Figure 5, supplementary Figure 4) – Specimen contains elements of the left and right limbs: four carpal elements of the right limb: the fused radiale and intermedium, ulnare, two other carpals (larger crescent shaped and smaller garlic shaped); fourteen elements of the right manus: all metacarpals, all phalanges of the digit I (phalanx and ungual), all phalanges of the digit II (1st, 2nd row phalanges and ungual), and probably all phalanges of the digit III (1st, 2nd, 3rd row phalanges and ungual); two carpal elements of the left limb: the fused radiale and intermedium and crescent shaped carpal; eight elements of the left manus: metacarpals I, II and IV, all phalanges of the digit I (1st row phalanx and ungual), all phalanges of the digit II (1st, 2nd row phalanges and ungual). The specimen should contain elements originally preserved in association and separated later during the preparation process. However, the preparation was done several years ago and it could be a mixture of several animals. Based on the state of preservation, it seems that elements of the right limb and probably the left fused radiale and intermedium belong to one animal. The elements of the left limb are much darker than those of the right, but they are of corresponding size to those of right. Exception is the crescent shaped carpal of the left limb, which have much different colour from other left hand elements corresponding more to those of the right, but it's size does not compare to similar element of the right hand.

- **ZPAL AbIII/ 257,1175, 2627** (supplementary Figure 5) - isolated humeri
- **ZPAL AbIII/1100/1, 1179, 2014, 3351** (supplementary Figure 6) - isolated ulnae
- **ZPAL AbIII/2106/2, 2106/4, 1628, 3322** (supplementary Figure 7) - isolated radii
- **ZPAL AbIII/2102** (supplementary Figure 8) - isolated metacarpal of the digit IV
- **ZPAL AbIII/267, 3352, 3353** (supplementary Figure 8) - isolated phalanges
