# Supplementary material for: Osteology of a forelimb of an aetosaur Stagonolepis olenkae (Archosauria: Pseudosuchia: Aetosauria) from the Krasiejów locality in Poland and its probable adaptations for a scratch-digging behavior
Source: PeerJ. 2018 Oct 2;6:e5595. doi: 10.7717/peerj.5595 (PMC6173166; doi:10.7717/peerj.5595)
Supplement: Appendix S2 [file peerj-06-5595-s002.pdf]

Appendix online 2. Measurements of studied forelimb bones of *Stagonolepis olenkae*

Letters in brackets refer to supplementary Figure 9

Table 1. Measurements of humeri

catalog no.	entire length (a) [cm]	width of the proximal head (b) [cm]	width of the shaft in the middle section (c) [cm]	width of the distal head (d) [cm]	thickness of the proximal head measured on top of articulation surface (e) [cm]	thickness of the shaft in the middle section (f) [cm]	thickness of the medial condyle (r) [cm]	thickness of the lateral condyle (s) [cm]	length of the deltopectoral crest (t) [cm]	perimeter of the shaft in the middle section [cm]
ZPAL AbIII/257	———	———	———	7.9	———	———	3.5	3.7	———	———
ZPAL AbIII/1175	24.0	12.7	3.1	7.9	3.7	2.3	3.3	4.0	7.5	8.7
ZPAL AbIII/2627	21.6	10.6	2.7		2.0	1.8	———	2.8	6.4	7.8

Table 2. Measurements of ulnae

catalog no.	entire length (a) [cm]	length to narrowing measured at the lateral side (h) [cm]	length to narrowing measured at the medial side (i) [cm]	length between coronoid process and the distal end of ulna (j) [cm]	width of the shaft in the proximal view (b) [cm]	width of the shaft in the middle section (c) [cm]	width of the shaft in the distal view (d) [cm]	thickness of the shaft in the proximal view (e) [cm]	thickness of the shaft in the middle section (f) [cm]	thickness of the shaft in the distal view (g) [cm]	length between the tip of olecranon process and the tip of coronoid process (k) [cm]	perimeter of the shaft in the middle section [cm]
ZPAL AbIII/1100/1	16.7	13.5	11.3	13.9	5.7	2.8	3.3	3.2	1.1	1.7	———	7.0
ZPAL AbIII/1179	18.2	13.2	11,3	15.0	6.0	3.0	3.3	3.9	1.3	1.7	———	7.5
ZPAL AbIII/2014	———	13.0	11.4	———	6.2	2.8	———	3.1	1.2	———	6.0	7.0
ZPAL AbIII/2407	17.5	———	———	14.4	5.2	———	3.1	3.1	1.2	1.8	5.7	———
ZPAL AbIII/3349/1	———	———	———	———	———	———	3.8	———	———	2.1	———	———
ZPAL AbIII/3349/2	———	———	———	———	———	———	3.8	———	———	1.9	———	———
ZPAL AbIII/3351	17.0	13.2	11.6	13.6	5.6	2.6	3.2	3.0	1.1	1.9	4.9	6.5

Table 3. Measurements of radii

catalog no.	entire length (a) [cm]	width of the shaft in the proximal view (b) [cm]	width of the shaft in the middle section (c) [cm]	width of the shaft in the distal view (d) [cm]	thickness of the shaft in the proximal view (e) [cm]	thickness of the shaft in the middle section (f) [cm]	thickness of the shaft in the distal view (g) [cm]	perimeter of the shaft in the middle section [cm]
ZPAL AbIII/1628	—	4.2	1.1	—	—	1.3	—	4.9
ZPAL AbIII/2106/2	—	—	—	3.0	—	—	1.7	—
ZPAL AbIII/2106/4	—	4.1	1.5	—	—	1.4	—	5.0
ZPAL AbIII/2407	14.8	4.2		3.3	—	1.6	1.6	4.7
ZPAL AbIII/3322	13.4	3.4	1.5	3.0	2.7	1.4	2.0	—
ZPAL AbIII/3349/1	—	—	—	—	—	—	1.9	—
ZPAL AbIII/3349/2	—	—	—	3.1	—	—	—	—

Table 4. Measurements of fused radiale and intermedium bones

catalog no.	entire width in proximal view (b) [cm]	length of the medial "radiale" side (l) [cm]	length of the lateral "intermedium" side (m) [cm]	thickness from the "radiale" side (n) [cm]	thickness from the "intermedium" side (p) [cm]
ZPAL AbIII/2071 left	3.6	1.6	1.8	2.9	1.8
ZPAL AbIII/2071 right	4.2	1.5	1.5	2.7	1.6
ZPAL AbIII/2407	4.3	1.6	—	2.6	—
ZPAL AbIII/3349/1	5.4	—	2.2	3.0	2.1
ZPAL AbIII/3349/2	5.2	2.0	—	3.0	—

Table 5. Measurements of ulnares

catalog no.	length [cm]	width [cm]	thickness [cm]
ZPAL AbIII/2071 right	0.8	2.1	1.7
ZPAL AbIII/2407	0.8	——	——
ZPAL AbIII/3349/1	1.0	2.3	2.0

Table 6. Measurements of other carpals

catalog no.	name	length [cm]	width [cm]	thickness [cm]
ZPAL AbIII/2071 right	distal carpal IV (larger, lunar shaped	1.2	2.2	1.0
	distal carpal III (smaller, pea shaped)	0.8	1.4	0.8
ZPAL AbIII/2071 left	distal carpal IV (larger, lunar shaped	1.5	2.1	1.1
ZPAL AbIII/3349/1	distal carpal IV (larger, lunar shaped)	1.2	2	1.8
	distal carpal III (smaller, pea shaped)	1.1	1.3	0.7

Table 7. Measurements of metacarpals

catalog no.	name	entire length (a) [cm]	width of the base in the proximal view (b) [cm]	width of the shaft in middle section (c) [cm]	width of the head in the distal view (d) [cm]	Thickness of the base in the proximal view (e) [cm]	thickness of the shaft in middle section (f) [cm]	thickness of the head in the distal view (g) [cm]
ZPAL AbIII/2071 right	metacarpal I	2.8	2.5	——	1.6	1.2	0.5	1.1
	metacarpal II	4.0	2.4	——	1.6	1.5	1.0	——
	metacarpal III	4.1	2.6	1.1	1.6	1.1	0.7	1.0
	metacarpal IV	3.9	2.0	1.0	1.5	——	0.7	1.0
	metacarpal V	2.9	1.8	0.9	1.4	1.2	0.7	0.7

Table 7 (cont.). Measurements of metacarpals

catalog no.	name	entire length (a) [cm]	width of the base in the proximal view (b) [cm]	width of the shaft in middle section (c) [cm]	width of the head in the distal view (d) [cm]	Thickness of the base in the proximal view (e) [cm]	thickness of the shaft in middle section (f) [cm]	thickness of the head in the distal view (g) [cm]
ZPAL AbIII/2071 left	metacarpal I	2.9	——	1.2	1.6	1.2	0.5	1.1
	metacarpal II	3.6	——	——	1.8	——	0.8	1.2
	metacarpal IV	3.9	2.2	1.1	1.4	1.9	0.7	1.0
ZPAL AbIII/2102	metacarpal IV	4.2	2.3	1.0	1.7	1.4	0.8	1.0
ZPAL AbIII/2407	metacarpal I	2.7	2.1	——	——	——	0.6	1.1
	metacarpal II	3.9	2.2	——	1.7	——	——	1.3
	metacarpal III	4.1	2.4	1.1	1.8	——	——	0.9
	metacarpal IV	3.9	——	1.1	1.9	——	——	1.0
	metacarpal V	2.8	1.9	1.0	——	1.0	0.7	0.7
ZPAL AbIII/3349/1	metacarpal I	3.2	2.8	——	2.1	1.3	——	0.9
	metacarpal II	4.6	3.2	——	2.0	1.2	——	1.1
	metacarpal III	4.4	2.2	1.7	2.3	1.4	1.4	0.9
	metacarpal IV	4.8	2.4	1.3	1.4	1.2	1.1	1.0
	metacarpal V	3.3	2.1	1.3	3.3	1.0	0.5	0.8
ZPAL AbIII/3349/2	metacarpal I	3.2	——	——	1.9	——	0.6	1.0
	metacarpal II	——	——	——	2.1	——	——	1.2
	metacarpal III	4.4	——	——	2.0	——	0.7	1.0
	metacarpal IV	——	——	1.1	1.8	——	0.7	1.0
	metacarpal V	3.0	——	——	——	——	0.5	0.7

Table 9. Measurements of phalanges

catalog no.	name	entire length (a) [cm]	width of the base in the proximal view (b) [cm]	width of the shaft in the middle section (c) [cm]	width of the head in the distal view (d) [cm]	thickness of the base in the proximal view (e) [cm]	thickness of the shaft in the middle section (f) [cm]	thickness of the head in the distal view (g) [cm]
ZPAL AbIII/267	2nd phalanx, digit III	1.4	1.3	1.0	1.2	0.9	0.5	0.7
ZPAL AbIII/2071 right	1st phalanx, digit I	1.4	1.4	———	1.2	———	———	1.0
	1st phalanx, digit II	1.8	———	1.0	1.3	———	0.7	1.0
	1st phalanx, digit III	1,7	1.5	———	1.4	1.5	———	0.8
	2nd phalanx, digit II	1.1	1.3	0.9	1.0	1.1	0.8	0.8
	2nd phalanx, digit III	1.5	1.3	0.8	1.2	1.1	0.6	0.6
	3rd phalanx, digit III	0.6	1.1	0.8	1.0	0.7	0.5	0.6
	Ungual, digit I	———	0.9	———	———	1.1	———	———
	Ungual, digit II	2.3	0.7	———	———	0.8<	———	———
	Ungual, digit III	1.7	0.5	———	———	0.6	———	———
ZPAL AbIII/2071 left	1st phalanx, digit I	1.4	1.3	1.0	1.2	———	———	1.0
	1st phalanx, digit II	2.0	1.7	1.0	1.4	1.4	0.7	0.9
	2nd phalanx, digit II	1.2	1.2	0.9	1.0	1.0	0.6	0.8
	Ungual, digit I	———	0.8	———	———	1.2	———	———
	Ungual, digit II	2.3	0.7	———	———	0.8	———	———
ZPAL AbIII/2407	1st phalanx, digit I	1.5	1.5	1.1	1.4	1.2	0.7	1.1
	1st phalanx, digit II	2.2	1.9	1.1	1.5	1.2	0.6	0.7
	1st phalanx, digit III	1.8	1.7	1.1	———	1.0	1.7	0.9
	1st phalanx, digit IV	1.8	1.4	1.1	1.4	1.1	———	0.8
	1st phalanx, digit V	1.5	———	———	1.0	0.8	0.5	0.6

Table 9 (cont.). Measurements of phalanges

catalog no.	name	entire length (a) [cm]	width of the base in the proximal view (b) [cm]	width of the shaft in the middle section (c) [cm]	width of the head in the distal view (d) [cm]	thickness of the base in the proximal view (e) [cm]	thickness of the shaft in the middle section (f) [cm]	thickness of the head in the distal view (g) [cm]
ZPAL AbIII/3349/1	1st phalanx, digit I	1.7	1.7	1.3	1.4	1.2	0.9	1.0
	1st phalanx, digit II	2.2	1.7	1.1	1.5	1.4	0.7	0.9
	1st phalanx, digit III	—	2.0	—	—	1.5	—	—
	1st phalanx, digit IV	1.9	1.7	1.0	1.4	1.2	0.6	0.7
	1st phalanx, digit V	1.4	1.4	—	1.2	0.9	0.4	0.5
	2nd phalanx, digit II	1.1	1.1	0.8	1.0	0.8	0.5	0.6
	2nd phalanx, digit IV	1.1	1.2	0.9	1.0	0.7	0.5	0.6
	3rd phalanx, digit IV	0.7	0.9	0.6	0.7	0.6	0.4	<0.5
	4th phalanx, digit IV	0.6	0.6	<0.6	0.5	<0.6	0.4	0.3
	Ungual, digit I	3.3	1.0	—	—	1.4	—	—
	Ungual, digit II	2.0	—	—	—	1.1	—	—
ZPAL AbIII/3349/2	1st phalanx, digit III	2.4	—	—	1.6	—	—	0.8
	1st phalanx, digit IV	—	—	—	1.4	—	—	0.7
	1st phalanx, digit V	1.7	1.2	—	1.2	—	—	—
	2nd phalanx, digit V	0.9	—	—	0.8	0.6	<0.4	0.4
ZPAL AbIII/3352	2nd phalanx, digit II	1.2	1.4	1.0	1.1	1.1	<0.6	<0.7
ZPAL AbIII/3353	3rd phalanx, digit III	0.6	0.8	<0.7	0.7	>0.5	0.5	<0.5
