# Supplementary material for: Osteology of a forelimb of an aetosaur Stagonolepis olenkae (Archosauria: Pseudosuchia: Aetosauria) from the Krasiejów locality in Poland and its probable adaptations for a scratch-digging behavior
Source: PeerJ. 2018 Oct 2;6:e5595. doi: 10.7717/peerj.5595 (PMC6173166; doi:10.7717/peerj.5595)
Supplement: Appendix S3 [file peerj-06-5595-s003.pdf]

Appendix online 3. Taphonomical notes on the forelimb specimens of *Stagonolepis olenkae*

The detailed studies on the taphonomy and origin of Krasiejów assemblage were conducted by Bodzioch and Kowal-Linka (2012) and general comments on the taphonomy and sedimentation of Krasiejów deposits are also included in the review of Krasiejów biota by Dzik and Sulej (2007).

Bodzioch and Kowal-Linka (2012) proposed that early diagenesis of aquatic animals from Krasiejów (*Paleorhinus*=*Parasuchus* and *Metoposaurus krasiejowensis*) occurred in a freshwater environment while early diagenesis of terrestrial *Stagonolepis olenkae* occurred in an arid terrestrial environment.

Rich bone-bearing horizons in Krasiejów contain bones of different animals which are usually mixed together (e.g., Dzik and Sulej 2007; Bodzioch and Kowal-Linka 2012). The character of this locality makes it often difficult to determine which remains belong to a single animal and due to that the bone elements not found in articulation must be interpreted as isolated. The bones are mostly preserved in three dimensional anatomical shape. They may be more or less deformed due to compaction of soft siltstone and mudstone, however the resulting changes of proportions of the bones are negligible and the original shape in most cases can be easily recognized.

The most common deformation among aetosaurs bones is dorso-ventral compression along their whole length. In the more fragile parts of the bones the external surface is often pushed inside the shaft forming deep dents. Those dents occur often in the long bones in the region behind the proximal and/or distal head. The pattern is often similar in the bones of the same type. In humeri, the dents occur usually on the ventral side behind the proximal head (ZPAL AbIII/2627, sup. Fig. 5B), in ulnae the dents occur usually on the ventral side behind the olecranon process (see spec. ZPAL Ab/III 2407, sup Fig. 1B; and ZPAL Ab/III 1179, 2014, 3351, sup. Fig. 6B–D), and in radii the dents occur usually in the regions behind the both proximal and distal heads at the dorsal and ventral side (ZPAL Ab/III 2407, sup. Fig. 1A; and ZPAL Ab/III 1628, 2106/4, 2106/2, 3222, sup. Fig. 7 A–D). In ulnae spec. ZPAL Ab/III 1100/1 and 1179 the proximal and distal heads are abraded missing olecranon processes and significant parts of the proximal sections. This damage could be an effect of decomposition in the terrestrial environment in the early stage of diagenesis before burial, according to hypothesis proposed by Bodzioch and Kowal-Linka (2012). In the specimens ZPAL Ab/III 2407, 3349/1, 3349/2 (sup. Fig. 1–3) the osteoderms are grouped closely with hand elements preserved in association. In living animals the osteoderms are attached to the skin within which they develop and grow (e.g., Vickaryous and Hall 2008). Their close arrange-

ment with articulated hand bones indicates early burial of the individuals with hands still covered by the skin. Dislocation of the bones in the specimens preserved in association (ZPAL Ab/III 2407, 3349/1, 3349/2, sup. Fig. 1–3) could be caused by the impact with the bottom of the water basin before burial. Similar arrangement of the bones was observed due to the impact with the bottom of the water pool in the taphonomical experiment conducted by Syme and Salisbury (2014) on the bodies of juvenile *Crocodylus porosus*.

References:

BODZIOCH, A. and KOWAL-LINKA, M. 2012. Unraveling the origin of the Late Triassic multitaxic bone accumulation at Krasiejów (S Poland) by diagenetic analysis. *Palaeogeography, Palaeoclimatology, Palaeoecology*, **346**, 25–36.

DZIK, J. and SULEJ, T. 2007. A review of the early Late Triassic Krasiejów biota from Silesia, Poland. *Paleontologia Polonica*, **64**, 3–27.

SYME, C. E. and SALISBURY, S. W. 2014. Patterns of aquatic decay and disarticulation in juvenile Indo-Pacific crocodiles (Crocodylus porosus), and implications for the taphonomic interpretation of fossil crocodyliform material. *Palaeogeography, Palaeoclimatology, Palaeoecology*, **412**, 108–123.

VICKARYOUS, M. K. and HALL, B. K. 2008. Development of the dermal skeleton in *Alligator mississippiensis* (Archosauria, Crocodylia) with comments on the homology of osteoderms. *Journal of morphology*, **269**, 398–422.
