# Supplementary material for: Osteology of a forelimb of an aetosaur Stagonolepis olenkae (Archosauria: Pseudosuchia: Aetosauria) from the Krasiejów locality in Poland and its probable adaptations for a scratch-digging behavior
Source: PeerJ. 2018 Oct 2;6:e5595. doi: 10.7717/peerj.5595 (PMC6173166; doi:10.7717/peerj.5595)
Supplement: Appendix S4 [file peerj-06-5595-s004.pdf]

Appendix online 4. Description of the method used to generate supplementary 3D models of the forelimb bones

Part 1. List of the programs used for generation of the 3D models, with the respective links to download them (if they are free to use):

1. **FastStone Photo Resizer, ver. 3.8**
   http://www.faststone.org/FSResizerDetail.htm
2. **VisualSFM**
   http://ccwu.me/vsfm/
3. **MeshLAB, 64bit, ver. 1.3.4 BETA**
   (currently newer version 2016.12 is available)
   http://www.meshlab.net/
4. **Texture Stitcher**
   (author: Michel Khazdan, Johns Hopkins University)
   http://www.cs.jhu.edu/~misha/Code/TextureStitcher/
5. **Daz Studio**
   https://www.daz3d.com/home
6. **Adobe Acrobat**

Part 2. Detail description of the method used to obtain 3D models:

1. Obtaining pictures for further processing

Photogrammetry allows reconstruction of the 3D shape of photographed objects from a set of photographs taken from different angles. The selected specimens of the aetosaur *Stagonolepis olenkae* were photographed with a 24,2 mega-pixels digital single-lens reflex (DSLR) Nikon D-3300 camera, mounted with AF-S DX Nikkor 18-105 mm f/3.5-5.6G ED VR lens. Each specimen was placed on a small desk (easy to walk around), at the centre of a piece of paper (A4 format) with continuous block of text printed (to ensure proper convergence of the orientation algorithm). Each specimen was photographed from two sides – dorsal and ventral, around 100 photos each. The models for both the ventral and the dorsal side were later scaled and digitally reassembled into a single object. Most of the images covered the entire surface of the specimen, and each overlapped with other photographs. The photographs were taken from 8 spots (in more or less regular intervals of approximately 45°), from different vertical angles (about 8, approximately form 0° to 90°), and from different distances. The images were acquired in the area with a constant, abundant, diffused light source to reduce glares and harsh shadows on the object. They were saved by the camera as .JPG files with the resolution of 6000x4000 px and later scaled down with the freeware program FastStone Photo Resizer to the resolution of 3200x2400 px, to speed up the photogrammetric processing.

2. Generation of dense point clouds in VisualSFM

Each dorsal and ventral sets of scaled images were imported into the freeware program VisualSFM (Wu 2011). VisualSFM allows to perform keypoint detection (unambiguous feature-points in each image) through SIFT algorithms (i.e. Scale Invariant Feature Transforms; Lowe 1999; Wu 2007), tie-points matching (matching points between pairs of photographs), sparse 3D points generation (Wu 2013; Wu *et al.* 2011) and finally to reconstruct high-resolution 3D point-clouds by dense correlation through CMVS-PMVS algorithms (i.e. Clustering Views for Multi-view Stereo and Patch Based Multi-view Stereo Software; Furukawa and Ponce 2010). For each specimen, two dense point clouds were generated (for dorsal and ventral surface) and saved as .PLY files.

3. Creation of the models in MeshLAB

Each of the generated files was imported into the freeware program MeshLAB (Cignoni *et al.* 2008). MeshLAB provides several tools for work with the 3D models, including cleaning, scaling, measuring, moving, and joining objects (Cignoni *et al.* 1998, Ranzgulia *et al.* 2012, Pietroni *et al.* 2010, Tarini *et al.* 2010, Vergne *et al.* 2010), as well as allows building triangulated meshes out of point clouds with Poisson Surface Reconstruction algorithm (Kazhdan and Hoppe 2013), and creates textures for the finished models (Ranzuglia *et al.* 2013). For each specimen, after manually cleaning unsubstantial points, two triangulated meshes and their respective textures were generated, and saved as .PLY files. Models of ventral and dorsal surface were aligned using the alignment tool (point based gluing) in MeshLAB. Then unsubstantial faces were manually cleaned, and both models were joined, using Surface Reconstruction: Poisson filter. The process, however, caused the loss of colour in generated model.

4. Color restoration with Texture Stitcher. Reduction, and scaling of the model in MeshLAB.

The coloration was restored using Laplace-Beltrami Operator algorithm, with Texture Stitcher program (Chang *et al.* 2009). Texture Stitcher allows to transfer colors from a few different meshes into the model generated from them. For the program to work, it requires transfer of color from the textures to vertices, and the coloration of the generated model is saved also into the vertices. The generated model is saved as a .PLY file. To properly save the coloration, the number of the vertices of the model needs to be increased a few times, and at this point the model is too big to be published. To reduce its size (reduce the number of vertices), the Quadric Edge Collapse Decimation filter was used. To preserve the coloration, the color from the vertices of the mesh exported by Texture Stitcher was saved to the texture generated for the smaller mesh. The model with smaller number of vertices and as-

signed texture was scaled to match the dimensions of the real specimen, and then exported as an .OBJ file. For each specimen an .OBJ file was created.

5. Export of the models into .PDF files.

The .OBJ files were imported into the freeware program Blender. This is a well-known tool allowing creation, management, and projection of 3D graphic. The models of each specimen were transformed in the program to match properly the X, Y, Z coordinate axis. Each model was than saved as an .OBJ file, and imported into Daz Studio. Daz Studio is another free to use program to manage and animate the models. The program allows to export textured models into .U3D files, which can be implemented into .PDF files. Exported .U3D files of each specimen were inserted into a .PDF file with Adobe Acrobat.

References:

CHUANG, M., LUO, L., BROWN, B. J., RUSINKIEWICZ, S. and KAZHDAN, M. 2009. Estimating the Laplace-Beltrami Operator by Restricting 3D Functions. *Computer graphics forum*, **28**, 1475–1484.

CIGNONI, P., ROCCHINI, C., and SCOPIGNO, R. 1998. Metro: Measuring error on simplified surfaces. *Computer Graphics Forum*, **17**, 167–174.

CIGNONI, P., CALLIERI, M., CORSINI, M., DELLEPIANE, M., GANOVELLI, F. and RANZUGLIA, G. 2008. Meshlab: an open-source mesh processing tool. *Sixth Eurographics Italian Chapter Conference*, pp. 129–136.

FURUKAWA, Y. and PONCE, J. 2010. Accurate, dense, and robust multiview stereopsis. *IEEE transactions on pattern analysis and machine intelligence*, **32**, 1362–1376. http://dx.doi.org/10.1109/TPAMI.2009.161.

KAZHDAN, M. and HOPPE, H. 2013. Screened poisson surface reconstruction. *ACM Transactions on Graphics (TOG)*, **32**, 29.

LOWE, D. G. 1999. Object recognition from local scale-invariant features. *Computer vision, 1999. The proceedings of the seventh IEEE international conference*, IEEE, Vol. 2, pp. 1150–1157.

PIETRONI, N., TARINI, M. and CIGNONI, P. 2010. Almost isometric mesh parameterization through abstract domains. *IEEE Transactions on Visualization and Computer Graphics*, **16**, 621–635.

RANZUGLIA, G., CALLIERI, M., DELLEPIANE, M., CIGNONI, P. and SCOPIGNO R. 2012. Efficient and Flexible Sampling with Blue Noise Properties of Triangular Meshes. *IEEE Transactions on Visualization and Computer Graphics*, **18**, 914–924.

RANZUGLIA, G., CALLIERI, M., DELLEPIANE, M., CIGNONI, P. and SCOPIGNO R. 2013. MeshLab as a complete tool for the integration of photos and color with high resolution 3D geometry data. *CAA 2012 Conference Proceedings*, pp. 406–416.

TARINI, M., PIETRONI, N., CIGNONI, P., PANOZZO, D. and PUPPO, E. 2010. Practical quad mesh simplification. *Computer Graphics Forum*, **29**, 407–418.

VERGNE, R., PACANOWSKI, R., BARLA, P., GRANIER, X. and SCHLICK, C. 2010. Radiance scaling for versatile surface enhancement. In *Proceedings of the 2010 ACM SIGGRAPH symposium on Interactive 3D Graphics and Games*, ACM, pp. 143–150.

WU, C. 2007. SiftGPU: A GPU Implemention of Scale Invariant Feature Transform (SIFT) [WWW Document].

WU, C. 2011. VisualSFM: A visual structure from motion system. [WWW document]. http://ccwu.me/vsfm/.

WU, C. 2013. Towards linear-time incremental structure from motion. In *3D Vision-3DV 2013, 2013 International conference on 3D Vison*, pp. 127–134. IEEE. http://dx.doi.org/10.1109/3DV.2013.25.

WU, C., AGARWAL, S., CURLESS, B. and SEITZ, S.M. 2011. Multicore bundle adjustment. *Computer Visualization Pattern Recognition (CVPR), 2011 IEEE Conference*, pp. 3057–3064, http://dx.doi.org/10.1109/CVPR.2011.5995552.
