# Supplementary material for: Osteology of a forelimb of an aetosaur Stagonolepis olenkae (Archosauria: Pseudosuchia: Aetosauria) from the Krasiejów locality in Poland and its probable adaptations for a scratch-digging behavior
Source: PeerJ. 2018 Oct 2;6:e5595. doi: 10.7717/peerj.5595 (PMC6173166; doi:10.7717/peerj.5595)
Supplement: Appendix S7 [file peerj-06-5595-s007.pdf]

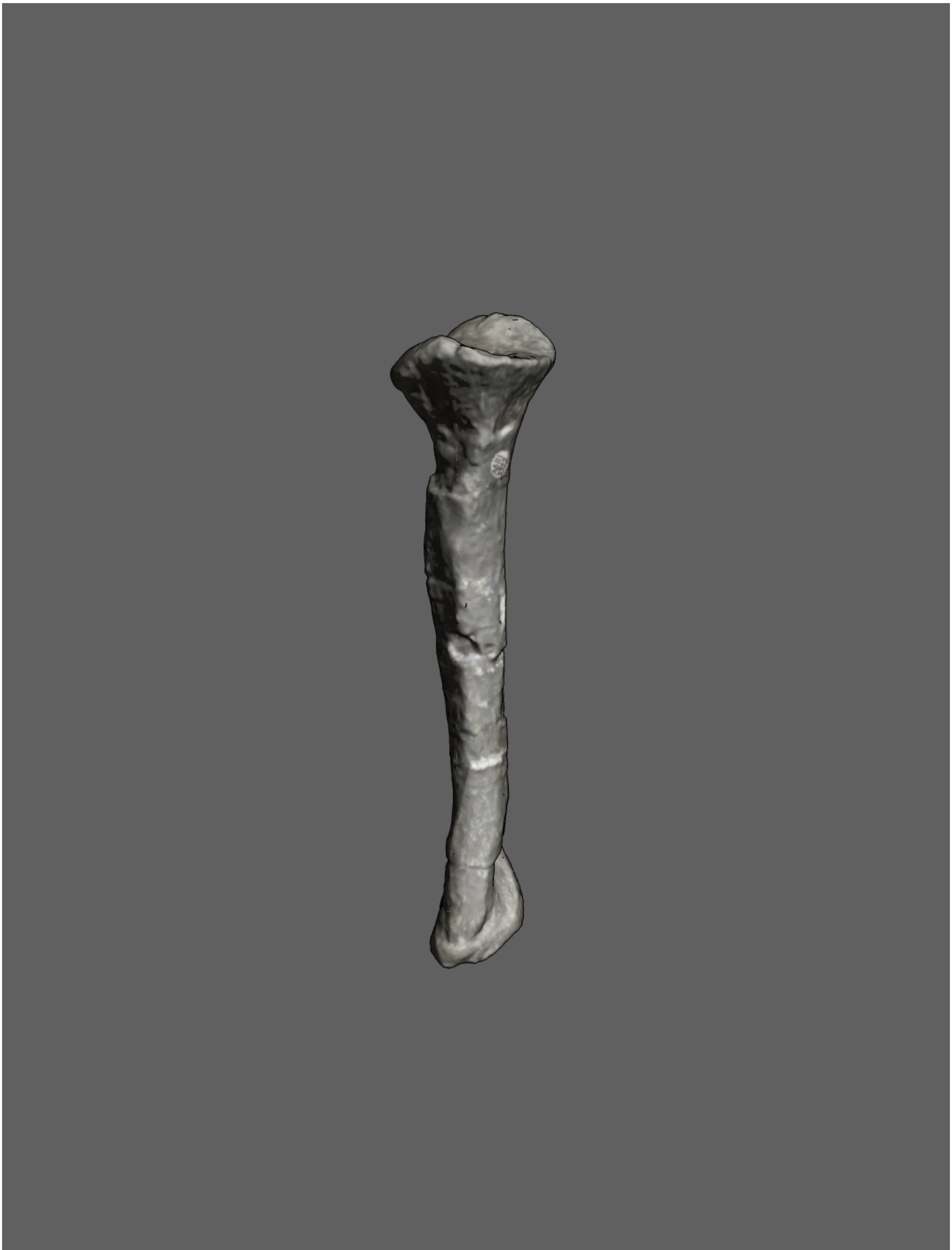

Appendix online 7. Interactive 3D model of the left radius ZPAL AbIII/3322 of the aetosaur *Stagonolepis olenkae* from the Krasiejów locality in Poland.
