# Supplementary material for: Osteology of a forelimb of an aetosaur Stagonolepis olenkae (Archosauria: Pseudosuchia: Aetosauria) from the Krasiejów locality in Poland and its probable adaptations for a scratch-digging behavior
Source: PeerJ. 2018 Oct 2;6:e5595. doi: 10.7717/peerj.5595 (PMC6173166; doi:10.7717/peerj.5595)
Supplement: Appendix S10 [file peerj-06-5595-s010.pdf]

# **Appendix online 10.** Detailed comparisons of forelimbs of the aetosaur *Stagonolepis olenkae* with other aetosaur species.

## Humerus

The overall shape and robustness of the humerus of *Stagonolepis olenkae* is most similar to large humeri of *Desmatosuchus smalli*, *Desmatosuchus spurensis* and *Longosuchus meadei* (Sawin 1947; Small 1985; Long and Murry 1995; Parker 2008; sup. Fig. 10A, B). In *S. olenkae*, like in *D. smalli*, *D. spurensis* and *L. meadei*, the humeral head is strongly medially expanded, with the internal tuberosity separated from the main articulation surface by a pronounced indentation, and the epicondyle is very widened medially, the shaft has similar outline in the proximal/distal view, with a distinct section of the humeral head and the base, and a relatively slim straight medial section (Sawin 1947; Small 1985; Long and Murry 1995). Similar conditions are present also in humerus of *Aetobarbakinoides brasiliensis* (Desojo et al. 2012; sup. Fig. 10H). In *Typothorax coccinarum* the humeral head is proportionally less expanded medially than in *S. olenkae*, and the internal tuberosity is not separated by any indentation from the main articular surface (it smoothly continues along the proximal edge of the head) (Long and Murry 1995; Martz 2002; Heckert et al. 2010; Sup. Fig. 10F). Also the middle section of the shaft appears to be proportionally longer than in *S. olenkae* (Long and Murry 1995; Martz 2002; Heckert et al. 2010). Based on the restoration of the humerus of *S. robertsoni* (fig. 14a, b in Walker 1961; sup. Fig. 10D) the general shape and proportions are almost identical to *S. olenkae*, but the humeral head in *S. robertsoni* is negligibly less expanded than in *S. olenkae* (*S. robertsoni* ratio of humeral head medio-lateral width to the entire length of humerus is about 0.58 and in *S. olenkae* 0.54) (Walker 1961; Parker 2016, 2018). The proximal articulation surface of the humerus in *S. robertsoni* is continuous, so there is no distinction of the internal tuberosity similar to *T. coccinarum* (Walker 1961; sup. Fig. 10D). The restored humerus of *S. robertsoni* does not have an olecranon fossa, as large as the humeri of *S. olenkae*, ZPAL AbIII/1175 and 257 (sup. Fig. 5A, C) (Walker 1961; sup. Fig. 10D). However, smaller humerus of *S. olenkae* ZPAL AbIII/2627 (sup. Fig. 5B) (and comparable in size to *S. robertsoni*) also does not have the olecranon fossa. Large humeri of "*Argentinosuchus bonapartei*" (spec. PVL 2091) and *Neoaetosauroides engaeus* have proximal heads strongly expanded medially like in *S. olenkae*, but there is no indentation between the internal tuberosity and proximal articulation like in *T. coccinarum* (Casamiquela 1960; Bonaparte 1971; Heckert and Lucas 2002; Desojo et al. 2012; sup. Fig. 10). The shafts of "*A. bonapartei*" and *N. engaeus* seem to be more robust than in *S. olenkae* (Casamiquela 1960; Bonaparte 1971; Heckert and Lucas 2002; Desojo et al. 2012; Sup. Fig. 10). The olecranon fossa present in large specimens of *S. olenkae*, can occur also in *N. engaeus* (Bonaparte

1971; sup. Fig. 10). In comparison to *S. olenkae*, the humeri of *Aetosauroides scagliai* (spec. PVL 2073) and *Polesinesuchus aurelioi*, apart from being much smaller (about 0.40 the length of the spec. ZPAL AbIII/1175 for *A. scagliai* and 0.25 for *P. aurelioi*) are less robust, their humeral heads and condyles are less expanded medially and laterally, and their deltopectoral crest is less developed (Heckert and Lucas 2002; Desojo and Ezcurra 2011; Roberto-da-Silva et al. 2014; sup. Fig. 10I, J). The humeri of *Aetosaurus ferratus* strongly differ from *S. olenkae* and other large aetosaurs in having slender proximal and distal portions, weakly developed deltopectoral crest and condyles of equal size (Schoch 2007; sup. Fig. 10K). The ectepicondylar groove in *S. olenkae* is fully exposed like in most aetosaurs, but unlike in *D. smalli*, *D. spurensis*, *L. meadei*, *T. coccinarum* and *N. engaeus* in which it is closed by bone and forms a foramen (Sawin 1947; Bonaparte 1971; Small 1985; Long and Murry 1995; Martz 2002; Desojo et al. 2013; sup. Fig. 10 A, B, F, G). *S. olenkae* have prominent supinator process. Marked supinator process is also present in *S. robertsoni* and *A. ferratus* (Walker 1961; Schoch 2007; sup. Fig. 10 D, K).

## Ulna

The ulna of *Stagonolepis olenkae* has a shape typical for most aetosaurs, having a well-developed proximal part with high olecranon process and the shaft being wide medio-laterally and flattened dorso-ventrally (e.g., Sawin 1947; Walker 1961; Schoch 2007; Roberto-Da-Silva et al. 2014; sup. Fig. 11D). The olecranon process is partially cartilaginous, which is suggested also for *Longosuchus meadei* (Sawin 1947) and *Polesinesuchus aureolii* (Roberto-Da-Silva et al. 2014) and was pointed as well for *S. olenkae* by Książkiewicz (2014). The height of the olecranon process in *S. olenkae* (around 0.2 in proportion to the entire length of the bone) is comparable to that of *Stagonolepis robertsoni*, *Typothorax antiquus*, *Typothorax coccinarum* and *Neoaetosauroides engaeus* (Sawin 1947; Walker 1961; Bonaparte 1971; Lucas et al. 2002; Heckert et al. 2010; sup. Fig. 11E–H). The olecranon process is higher than in *S. olenkae*, in *L. meadei* and *D. smalli* (being around 0.25 of the entire ulna length in *L. meadei* and 0.3 in *D. smalli*) (based on fig. 4c, d in Sawin 1947; and fig. 8 c, d in Small 1985; sup. Fig. 11A, B). In *Aetosaurus ferratus*, *P. aureolii*, and *Aetosauroides scagliai* (PVL 2073) the olecranon process is shorter than in *S. olenkae* (being around of the entire ulna length 0.15 in *A. ferratus*, 0.14 in *A. scagliai* and 0.10 in *P. aureolii* (based on fig. 3-3, 4 in Heckert and Lucas 2002; fig. 10G in Schoch 2007; fig. 21 in Roberto-Da-Silva et al. 2014; sup. Fig. 11I–K). The coronoid process in *S. olenkae* is elongated medially, pronounced and with a sharp end (spec. ZPAL AbIII 2014, 3351, fig. 6C, D), similar to *D. smalli* and "*Argentinosuchus bonapartei*" (spec. PVL 2091) (Small 1985; Heckert and Lucas 2002). In *L. meadei*, *T. coccinarum*, *T. antiquus*, and *S. robertsoni* the coronoid process

is also elongated medially, but its end is smooth (Sawin 1947; Walker 1961; Lucas et al. 2002; Heckert et al. 2010; sup. Fig. 11B, E–H). In *A. ferratus*, *P. aureolii*, and *A. scagliai* (PVL 2073) the coronoid process is much less developed than in *S. olenkae* (Heckert and Lucas 2002; Schoch 2007; Desojo and Ezcurra 2011; Roberto-Da-Silva et al. 2014; sup. Fig. 11I–K).

## Radius

The radius of *Stagonolepis olenkae* is similar to known aetosaurs in having a straight shaft with equally expanded proximal and distal ends (e.g., Sawin 1947; Walker 1961; Lucas et al. 2002; Schoch 2007). The level of expansion of the proximal and distal end in *S. olenkae* (in proportion to the entire length of the radius) is comparable to *Longosuchus meadei*, *Stagonolepis robertsoni*, *Typothorax antiquus*, and *Typothorax coccinarum* (Sawin 1947, Walker 1961; Lucas et al. 2002; Heckert et al. 2010; sup. Fig. 11E, G, H). In *Aetosaurus ferratus* and *Aetosauroides scagliai* (spec. PVL 2073) the proximal and distal ends of radius are less expanded (Heckert and Lucas 2002; Schoch 2007; Desojo and Ezcurra 2011; Sup. Fig. 11I, K). The square cross-section of the shaft of the radius of *S. olenkae* is a character that distinguishes it from other known aetosaurs, including *S. robertsoni*, *L. meadei*, *A. ferratus*, and *T. antiquus*, in which the cross section is oval (Sawin 1947; Walker 1961; Schoch 2007; Lucas et al. 2002).

## Carpus

*Stagonolepis olenkae* is one of few aetosaurs with known carpal elements, along with the *Aetosaurus ferratus*, *Longosuchus meadei*, *Stagonolepis robertsoni*, and *Typothorax coccinarum* (Sawin 1947; Walker 1961; Schoch 2007; Lucas and Heckert 2011; sup. Fig. 12). The number of carpals in *S. olenkae* is four, which is similar to *L. meadei* and *T. coccinarum* (MCZ 1487) (Sawin 1947; Lucas and Heckert 2011). Książkiewicz (2014) speculated that in *S. olenkae* could be up to five carpals, but there is no specimen with more than four carpal bones preserved. In *S. robertsoni* and *A. ferratus* two carpals are recognizable, but in some individuals of *A. ferratus* more could be preserved (Walker 1961, Schoch 2007). In *S. olenkae*, *S. robertsoni*, *A. ferratus*, and *L. meadei* the dominant carpal bone is the large fused radiale and intermedium (Sawin 1947, Walker 1961, Schoch 2007). Książkiewicz (2014) described one specimen of *S. olenkae* (UOBS 02830) with those two bones separated, but as he suggested, the fused radiale and intermedium in this case is most likely broken. In *Typothorax coccinarum* (MCZ 1487) the carpal bones are of comparable size, but the rounded bone opposite to the radius seems to be little larger than others and its shape is

corresponding to the lateral side of the fused radiale and intermedium of *S. olenkae*, therefore it could also be a fused radiale and intermedium (based on fig. 4a, b in Lucas and Heckert 2011). The fused radiale and intermedium in *S. robertsoni* seems to be smaller than its homologue in *S. olenkae*, as it reaches only to the metacarpal III, while in *S. olenkae* it is adjacent to the metacarpal III and also part of the metacarpal IV (Walker 1961; sup. Fig. 12F).

I propose the arrangement of the carpal elements in *Stagonolepis olenkae* in two rows, first consisting of the fused radiale and intermedium with the ulnare and the second consisting of two distal carpals. In *Longosuchus meadei*, the only other aetosaur species in which the arrangement of carpals is proposed, the fused radiale and intermedium forms a single row with the other three carpals (Sawin 1947; sup. Fig. 12A). However Sawin (1947), when preparing his restoration of *L. meadei*, did not have at his disposal any articulated carpus and was able to identify only the fused radiale and intermedium, already known to occur in *A. ferratus*. He wrote that the arrangement of the other carpal elements in one row was only his best assumption (Sawin 1947). Książkiewicz (2014) also suggest arrangement of the carpal bones in a single row for *S. olenkae*, but he as well did not have any specimens with articulated carpus. The arrangement of carpals in two rows for *S. olenkae* is strongly supported by ZPAL AbIII/2407 (sup. Fig. 1), in which the carpus and hand bones are preserved the closest to their anatomical position among studied specimens. In this specimen the fused radiale and intermedium form one row with the ulnare, and another (distal) carpal is preserved in front of them, towards metacarpals. Similar condition could be observed also in the spec. ZPAL AbIII/3349/1 (sup. Fig. 2), in which the distal carpals are preserved in front of the ulnare, opposite to the third metacarpal. However, in this specimen the fused radiale and intermedium is not preserved in articulation and it is not certain how much the position of other carpals changed in respect to their original setting. The arrangement of carpals in two rows is also recognized in *Postosuchus alisonae* – rauisuchid closely related to aetosaurs (Peyer et al. 2008). The longer side of the ulnare in the spec. ZPAL AbIII/2407 (sup. Fig. 1) faces the metacarpals and forearms. In this setting, the medial side of the ulnare seems to be disproportionally narrow in comparison to the corresponding surface on the fused radiale and intermedium (manuscript Fig. 6C). Nevertheless, in ornithosuchians, other basal archosaurs closely related to aetosaurs, a huge disproportion in size between the radiale and the intermedium is possible (Bonaparte 1971; Von Baczko and Ezcurra 2013). The carpus of modern crocodilians consist of both bony and cartilaginous elements (e.g., Buscalioni et al. 1997) and since the preserved carpal bones does not fit perfectly with the metacarpals and long

bones, it is probable that also in *S. olenkae* the carpus could consist of cartilage elements or at last the amount of cartilage covering the bones was significant.

## Manus

Palm bones, similarly as carpals, are also known for only a few aetosaurs apart of *S. olenkae*. The others are *Aetosaurus ferratus, Longosuchus meadei*, *Stagonolepis robertsoni*, *Typothorax coccinarum* (Sawin 1947; Walker 1961; Schoch 2007; Lucas and Heckert 2011; Sup. Fig. 12). The metacarpals and phalanges of *S. olenkae* are robust, short, and dorso-ventrally flattened similarly to *L. meadei*, *S. robertsoni*, and *T. coccinarum* (Sawin 1947; Walker 1961; Lucas and Heckert 2011; sup. Fig. 12A, D, F). The manus of *A. ferratus* seems to be more slender than that of *S. olenkae* and other known aetosaurs (Schoch 2007; sup. Fig. 12C). Unfortunately, none of the described specimens of aetosaur manus have complete set of phalanges, so the phalangeal formula proposed for aetosaurs remains hypothetical (Sawin 1947, Walker 1961, Schoch 2007, this study). In *S. olenkae* none of the studied specimens preserve all of the distal phalanges of digits IV and V. Considering the number of phalanges in the preserved specimens and their size, the most simple phalangeal formula for S. olenkae is 2-3-4-5-3, the same as for *S. robertsoni* (Walker 1961). The same number of phalanges was suggested by Książkiewicz (2014). The phalangeal formula proposed for *L. meadei* (2-3-4-5-2) cannot be applied to *S. olenkae* because in the spec. UOBS 02834 described by Ksiażkiewicz (2014) the fifth digit has two phalanges even though the ungual is not preserved. Need to be mentioned that the phalangeal formula proposed by Sawin (1947) could be a mistake, because the restoration of manus of *L. meadei* (fig. 3c in Sawin 1947) shows two phalanges and small ungual on the fifth digit. Thus based on the Sawin's restoration, the phalangeal formula of *L. meadei* should be 2-3-4-5-3, which is consistent with proposed formulas of *S. olenkae* and *S. robertsoni* (Sawin 1947; sup. Fig. 12A, B, F). In *A. ferratus* the phalangeal formula cannot be determined (Schoch 2007). The claw-like unguals in *S. olenkae* are present on at least first three digits (based on spec. ZPAL AbIII/2071, Fig. 4), similarly to *S. robertsoni* (Walker 1961; sup. Fig. 12F). In *L. meadei* they are preserved on the first two digits (Sawin 1947; Sup. Fig. 12A). In *S. olenkae* the size of unguals decreases in regular pattern with the second ungual being one–third the length of the first, and the third being one-third the length of the second. Middle digits (II, III, IV) are almost of equal length in *S. olenkae*, while the external digits are much shorter, about 0.75 the length of the middle digits. The same pattern of digit length characterizes *S. robertsoni*, but Walker (1961) suggested that the digit III was probably the longest one and the same proportions are also established for *L. meadei* and *T. coccinarum*, as both Sawin (1947) and Lucas and Heckert (2011) indicated the digit III is a little longer than the II and IV. Furthermore, the lengths of

digits in *T. coccinarum* are also inferred to be III>II>IV>I>V, based on the probable correspondence of tracks of *Brachychirotherium* to this species (Lucas and Heckert 2011). In *S. olenkae*, as in *S. robertsoni*, *L. meadei*, and *T. coccinarum*, the first digit is the most robust and the fifth digit is the tiniest and reduced (Sawin 1947, Walker 1961, Lucas and Heckert 2011, Książkiewicz 2014, this study). It is probable that in *S. olenkae* the fifth digit was not functional in stepping, as was probably the case in *T. coccinarum* (Lucas and Heckert 2011). The fifth metacarpal of *S. olenkae* in the spec. ZPAL AbIII/3349 (sup. Fig. 2), 3350 (sup. Fig. 3) is dorso-ventrally flattened, very wide (as wide as the metacarpal IV) and its proximal head is not distinct, however in the spec. ZPAL AbIII/2071 (sup. Fig. 4) and 2407 (sup. Fig. 1) the fifth digit is thin, oval in cross section and with a distinct proximal head. The latter conditions are similar to *S. robertsoni*, *L. meadei*, and *T. coccinarum* (Sawin 1947, Walker 1961, Lucas and Heckert 2011). Differences in shape of the fifth metacarpal in S. olenkae may be due to intraspecific variation, dimorphism or ontogeny, since the spec. ZPAL AbIII/3349 (sup. Fig. 2) and 3350 (sup. Fig.3) belong to larger individuals than ZPAL AbIII/2071 and 2407.

## Dermal armour

Probably the entire arm, carpus, and hand of *Stagonolepis olenkae* were covered by osteoderms at least dorsally. The osteoderms covering the arms and forearms are larger than those of the manus and carpus, and it is probable that they were arranged in rows, similar to *Aetosaurus ferratus* and *Typothorax coccinarum* (Schoch 2007; Heckert et al. 2010). The presence of osteoderms on arms and forearms is also reported for *Stagonolepis robertsoni* (Walker 1961) and it is probable that they were also present in "*Argentinosuchus bonapartei*", since the appendicular osteoderms are found in the sediment associated with humerus (PVL 2091) (Heckert and Lucas 2002). *S. olenkae* is the only known aetosaur in which the osteoderms cover the carpus and manus region. In modern crocodiles each osteoderm corresponds to a keratinous scale of similar shape (Burns et al. 2013). The presence of such scales can be expected also in aetosaurs.

## Increase of size as a probable explanation of some variability in aetosaurs forelimbs

The studies conducted by Bonnan *et al.* (2013) on the changes of the shape of long bones of archosaurs and mammals, showed that expansion of long bone joints, associated with an increase of cartilage volume, are typical features connected with the escalation of size in archosaurs. The increase of bone robustness, increase of transverse length of distal and proximal heads of the humerus, as well as increase of length of the olecranon process, and expansion of proximal and distal parts of the radii, correlated with an increase of the animal

size can be easily observed in transition from small to large aetosaur species (Sawin 1947; Walker 1961; Small 1985; Lucas et al. 2002; Heckert and Lucas 2002; Martz 2002; Schoch 2007; Parker 2008; Desojo and Ezcurra 2011; Desojo et al. 2012; Lucas and Heckert 2011; Roberto-Da-Silva et al. 2014; sup. Fig. 10, 11). Considering the research of Bonnan *et al.* (2013) I think that evolutionary increase of size might be a major factor determining at least the above mentioned variability between aetosaurs, but further studies are needed to additionally support this statement.

**References:**

AGASSIZ, L. 1844. Monographie des Poissons Fossiles du Vieux Gres Rouge ou Systeme Edevonien (Old Red Sandstone) des Iles Britanniques et de Russie: Jent Et Gassman, Neuchatel, 171 pp.

BONAPARTE, J. F. 1969. Los tetrápodos triásicos de Argentina. In *Simposio Internacional sobre Estratigrafía y Paleontología del Gondwana,* **1**, 307–325.

BONNAN, M. F., WILHITE, D. R., MASTERS, S. L., YATES, A. M., GARDNER, C. K., and AGUIAR, A. 2013. What lies beneath: sub-articular long bone shape scaling in eutherian mammals and saurischian dinosaurs suggests different locomotor adaptations for gigantism. *PloS One*, **8**, e75216, https://doi.org/10.1371/journal.pone.0075216.

BURNS, M. E., VICKARYOUS, M. K., and CURRIE, P. J. 2013. Histological variability in fossil and recent alligatoroid osteoderms: systematic and functional implications. *Journal of Morphology*, **274**, 676–686.

BUSCALIONI, A. D., ORTEGA, F., RASSKIN-GUTMAN, D. and PÉREZ-MORENO, B. P. 1997. Loss of carpal elements in crocodilian limb evolution: morphogenetic model corroborated by palaeobiological data. *Biological Journal of the Linnean Society,* **62**, 133–144.

CASE, E. C. 1920. Preliminary description of a new suborder of phytosaurian reptiles with a description of a new species of *Phytosaurus*. *The Journal of Geology*, **28**, 524–535.

CASAMIQUELA, R. M. 1960. Notica preliminar sobre dosnuevos estagonolepoideos Argentinos. *Ameghiniana*, **2**, 3–9.

COPE, E. C. 1875. Report on the geology of that part of northwestern New Mexico examined during the field-season of 1874: Annual Report Upon the Geographical Explorations West of the 100th Meridian [Wheeler Survey], Appendix LL, Annual Report Chief of Engineers for 1875, Washington, pp. 61–97 of separate issue, pp. 981–1017 of full report.

BONAPARTE, J. F. 1971. Los tetrapodos del sector superior de la Formacion los Colorados, La Rioja, Argentina (Triasico Superior). Part I. *Opera Lilloana*, **22**, 1–188.

DESOJO, J. B. and EZCURRA, M. D. 2011. A reappraisal of the taxonomic status of *Aetosauroides* (Archosauria, Aetosauria) specimens from the Late Triassic of South America and their proposed synonymy with *Stagonolepis*. *Journal of Vertebrate Paleontology*, **31**, 596–609.

DESOJO, J. B., EZCURRA, M. D. and KISCHLAT, E. E. 2012. A new aetosaur genus (Archosauria: Pseudosuchia) from the early Late Triassic of southern Brazil. *Zootaxa*, **3166**, 1–33.

DESOJO, J. B., HECKERT, A. B., MARTZ, J. W., PARKER, W. G., SCHOCH, R. R., SMALL, B. J. and SULEJ, T. 2013. Aetosauria: a clade of armoured pseudosuchians from the Upper Triassic continental beds. *In* NESBITT, S. J., DESOJO, J. B. and IRMIS, R. B. (eds) 2013. *Anatomy, Phylogeny and Palaeobiology of Early Archosaurs and their Kin. Geological Society, London, Special Publications,* **379**, 203–239.

FRASS, O. 1877. *Aëtosaurus ferratus*, die gepanzerte Vogelechse aus aus dem Stubensandstein bei Stuttgart. *Jahreshefte des Vereins für vaterländische Naturkundein Württemberg,* **33**, 1–21.

HECKERT, A. B. and LUCAS, S. G. 2002. South American occurrences of the Adamanian (Late Triassic: latest Carnian) index taxon Stagonolepis (Archosauria: Aetosauria) and their biochronological significance. *Journal of Paleontology*, **76**, 852–863.

HECKERT, A. B., RINEHART, L. F., CELESKEY, M. D., SPIELMANN, J. A. and HUNT, A. P. 2010. Articulated skeletons of the aetosaur *Typothorax coccinarum* Cope (Archosauria: Stagonolepididae) from the Upper Triassic Bull Canyon Formation (Revueltian: early-mid Norian), eastern New Mexico, USA. *Journal of Vertebrate Paleontology*, **30**, 619–642.

HUNT, A. P. and LUCAS, S. G. 1990. Re-evalution of "*Typothorax*" *meadei*, a Late Triassic aetosaur from the United States. *Paläontologische Zeitschrift*, **64**, 317–328.

KSIĄŻKIEWICZ, K. 2014. Morfo-funkcjonalna analiza szkieletu postkranialnego opancerzonego archozaura rodzaju *Stagonolepis* z późnego triasu polski. Unpublished Doctoral's thesis. Uniwersity of Wrocław, Wrocław, 147 pp.

LONG, R. A. and MURRY, P. A. 1995. *Late Triassic (Carnian and Norian) Tetrapods from the Southwestern United States: Bulletin 4 (Vol. 4), Chapter 6*, New Mexico Museum of Natural History and Science, Albuquerque, pp. 66–116.

LUCAS, S. G. and HECKERT, A. B. 2011. Late Triassic aetosaurs as the trackmaker of the tetrapod footprint ichnotaxon *Brachychirotherium*. *Ichnos*, **18**, 197–208.

LUCAS, S. G., HECKERT, A. B. and HUNT, A. P. 2002. A new species of the aetosaur *Typothorax* (Archosauria: Stagonolepididae) from the Upper Triassic of east-central New Mexico. *New Mexico Museum of Natural History and Science Bulletin*, 21: 221–233.

MARTZ, J. W. 2002. The morphology and ontogeny of *Typothorax coccinarum* (Aarchosauria, Stagonolepididae) from the upper triassic of the american southwest. Unpublished master's thesis. Texas Tech University, Lubbock, Texas, 279 pp.

PARKER, W. G. 2005. A new species of the Late Triassic aetosaur *Desmatosuchus* (Archosauria: Pseudosuchia). *Comptes Rendus Palevol*, **4**, 327–340.

PARKER, W. G. 2008. Description of new material of the aetosaur *Desmatosuchus spurensis* (Archosauria: Suchia) from the Chinle Formation of Arizona and a revision of the genus *Desmatosuchus. PaleoBios*, **28**, 1–40.

PARKER, W. G. 2016. Revised phylogenetic analysis of the Aetosauria (Archosauria: Pseudosuchia); assessing the effects of incongruent morphological character sets. *PeerJ*, **4**, e1583. DOI 10.7717/peerj.1583.

PARKER, W. G. 2018. Redescription of *Calyptosuchus* (*Stagonolepis*) *wellesi* (Archosauria: Pseudosuchia: Aetosauria) from the Late Triassic of the Southwestern United States with a discussion of genera in vertebrate paleontology. *PeerJ*, **6**, e4291. DOI: 10.7717/peerj.4291.

PEYER, K., CARTER, J. G., SUES, H. D., NOVAK, S. E. and OLSEN, P. E. 2008. A new suchian archosaur from the Upper Triassic of North Carolina. *Journal of Vertebrate Paleontology*, **28**, 363–381.

ROBERTO-DA-SILVA, L., DESOJO, J. B., CABREIRA, S. F., AIRES, A. S., MUELLER, R. T., PACHECO, C. P. and DIAS-DA-SILVA, S. 2014. A new aetosaur from the Upper Triassic of the Santa Maria Formation, southern Brazil. *Zootaxa*, **3764**, 240–278.

SAWIN, H. J. 1947. The pseudosuchian reptile *Typothorax meadei*. *Journal of Paleontology*, **21**, 201–238.

SCHOCH, R. R. 2007. Osteology of the small archosaur *Aetosaurus* from the Upper Triassic of Germany. *Neues Jahrbuch für Geologie und Paläontologie, Abhandlungen*, **246**, 1–35.

SMALL, B. J. 1985. The Triassic thecodontian reptile Desmatosuchus: osteology and relationships. Unpublished Master's thesis, Texas Tech University, Lubbock, Texas, USA, 83 pp.

SULEJ, T. 2010. The skull of an early Late Triassic aetosaur and the evolution of the stagonolepidid archosaurian reptiles. *Zoological Journal of the Linnean Society*, **158**, 860–881.

VON BACZKO, M.B. and EZCURRA, M. D. 2013. Ornithosuchidae: a group of Triassic archosaurs with a unique ankle joint. *In* NESBITT, S. J., DESOJO, J. B. and IRMIS, R.B. (eds) 2013. *Anatomy, Phylogeny and Palaeobiology of Early Archosaurs and their Kin. Geological Society, London, Special Publications*, **379**, 187–202.

WALKER, A. D. 1961. Triassic reptiles from the Elgin area: *Stagonolepis*, *Dasygnathus* and their allies. *Philosophical Transactions of the Royal Society B: Biological Sciences*, **244**, 103–204.
