# Supplementary material for: Osteology of a forelimb of an aetosaur Stagonolepis olenkae (Archosauria: Pseudosuchia: Aetosauria) from the Krasiejów locality in Poland and its probable adaptations for a scratch-digging behavior
Source: PeerJ. 2018 Oct 2;6:e5595. doi: 10.7717/peerj.5595 (PMC6173166; doi:10.7717/peerj.5595)
Supplement: Appendix S11 [file peerj-06-5595-s011.pdf]

Appendix online 11. Explanation of adaptive characteristics for scratch-diggers

Scratch-digging is the method of digging in which by alternate flexing and extending the limbs the animal cuts and loosens the soil with its claws and pushes or flings it to the rear (=for example as a dog trying to bury the bone) (Hildebrand 1988). The most powerful modern scratch-diggers are armadillos, the pangolins, and the aardvark, but several other animals dig this way, including some frogs, some tortoises, some birds, fossorial marsupials, most badgers, a ratel, ground squirrels and variety of other mammals (some with scant structural adaptations for it, like canids) (Hildebrand 1983, 1988).

The digging requires enormous strength, therefore the limbs of specialized scratch-diggers are modified to be able to apply a great force against the hard, solid substrate (Hildebrand 1988). During the process, most scratch-diggers extend the forefeet to the earth and then draw the claws downward, toward or under the body (Hildebrand 1988). In this case the arm works as a lever in which the in-force (F_i) is applied to the olecranon process being the lever arm (power arm) and the out-force (F_o) is produced at the end of the hand, while the forearm and the hand (with exception of the olecranon process) are the out lever arm (load arm) (Hildebrand 1988). Considering that $F_o=\frac{Fi*li}{lo}$ (l_i - length of the power arm, l_o - length of the load arm), there are three major ways to increase the out-force (F_o) (Hilderand 1988). The first way is to reduce the length of the out lever arm (l_o). As a result, the limbs of specialized diggers in general have relatively short distal segments: the radius is shorter than the humerus (low brachial index), the manus is shorter than the radius (exclusive of terminal phalanges and their claws), the carpus is short, the metacarpals and phalanges are short and wide, with proximal phalanges often broader than long (Coombs 1983, Hildebrand 1983, 1988, Heckert *et al.* 2010). The second way to increase the out-force (F_o) is to increase the related in-lever (l_i). Due to that, the muscles used in digging tend to insert far from the joints they turn (Hildebrand 1988). Accordingly, the insertions of the deltoid muscles (deltoid crest) in diggers commonly extends more than halfway of the humerus, the median epicondyle of the humerus is wide (feature of all scratch-diggers, origin of forearm pronator and manual flexors), the origin of the supinator muscle on the humerus (the supinator crest) is proximally extended, and the pisiform is elongated (insertion of the flexor carpi ulnaris muscle) (Hildebrand 1988, Coombs 1983, Heckert *et al.* 2010). For the same reason, the olecranon process of ulna in diggers is usually long (Coombs 1983, Hildebrand 1988, Heckert *et al.* 2010). The Third way to increase the out-force (F_o) is to increase the in-force (F_i). Consequently the muscles connected to the digging behaviour are exceptionally well-developed in diggers, and

to accommodate such muscles, their insertions and origins are enlarged (Hildebrand 1988). As a result, the bones of diggers are usually rugged and rough (Hildebrand 1988). The medial epicondyle of humerus (origin of flexors of digits) and the deltoid crest (insertion of the deltoids) are particularly prominent, as well as the acromion process of the scapula (origin of deltoid musculature) and posterior angle of a scapula (origins of the teres major and the long head of the triceps) (Hildebrand 1988, Coombs, 1983, Heckert *et al.* 2010).

Application of a relatively great force puts the joints of the diggers under much higher stress then in other animals, therefore they are usually better adapted in several ways to resist hyperextension, dislocation, and counterproductive deflection (Hildebrand 1983). The motion of joints that in less specialized animals allows for moves in several planes, in digging animals tend to become limited to a single plane: the wrist joint becomes hinge-like (permitting only flexion and extension), the head of the humerus may has a greater radius of curvature in horizontal than in vertical plane (limiting or preventing adduction and abduction), and the acromion process is long (also limiting adduction and abduction) (Hildebrand 1983). Likewise, joints that usually permit motion, are modified to allow little or no motion: joints between the phalanges and joints between the digits and metacarpals may be flat-ended, V-shaped, multiangled or provided with a peg and a socket, and the surfaces of the distal phalanges cover the surfaces of the proximal ones (preventing sliding movements) (Hildebrand 1983). Rigidity may also be achieved by loss and fusion of bones (Hildebrand 1983). For example, the number of phalanges preceding the unguals decreases in digging mammals (usually to two or one), in some few digits may be modified into digging tools and others may be lost or reduced, the metacarpals and proximal phalanges may be fused, and some elements of the carpus may be horizontally fused (Hildebrand 1983, 1988). Furthermore, joints that normally provide for motion in one plane tend to become strengthened against dislocation: distal ends of the ones preceding the terminal phalanges are enlarged with relatively great radius of curvature (increasing the surface contact between the bones), palm bones develop structures that resist dislocation (for example vertical splines and compatible grooves in metacarpals and phalanges), large sesamoid bones may be present under the joints (their presence functionally bond the distal phalanges to the proximal ones, they also brace joints against motion, and guide or serve as attachments for tendons) (Hildebrand 1983). Diggers also commonly have bony stops between phalanges, and metacarpals and phalanges, that work as a passive mechanism to prevent hyperextension of hinge joints (Hildebrand 1983, 1988).

Scratch-diggers often need to excavate in a dry, compacted soil, therefore in addition to being able to produce great force, they usually have impressive elongated terminal phalanges changed into blade-like or pick-like structures, and covered by strong keratin sheath (Hildebrand 1983, 1988). Claws allow them to concentrate the delivered force into small, restricted area which makes it much easier to break the hard, consolidated substrate (Hildebrand 1988).

References:

COOMBS, M. C. 1983. Large mammalian clawed herbivores: a comparative study. *Transactions of the American Philosophical Society*, **73**, 1–96.

HECKERT, A. B., RINEHART, L. F., CELESKEY, M. D., SPIELMANN, J. A. and HUNT, A. P. 2010. Articulated skeletons of the aetosaur *Typothorax coccinarum* Cope (Archosauria: Stagonolepididae) from the Upper Triassic Bull Canyon Formation (Revueltian: early-mid Norian), eastern New Mexico, USA. *Journal of Vertebrate Paleontology*, **30**, 619–642.

HILDEBRAND, M. 1983. Digging of quadrupeds. *In* HILDEBRAND, M., BRAMBLE, D. M., LIEM, K. F. and WAKE, D. B. (eds.), *Functional Vertebrate Morphology*. Harvard University Press, Cambridge, Massachusetts, pp. 89–109.

HILDEBRAND, M. 1988. *Analysis of Vertebrate Structure*. *Third Edition*. John Wiley and Sons, New York, 701 pp.
