# Supplementary material for: Osteology of a forelimb of an aetosaur Stagonolepis olenkae (Archosauria: Pseudosuchia: Aetosauria) from the Krasiejów locality in Poland and its probable adaptations for a scratch-digging behavior
Source: PeerJ. 2018 Oct 2;6:e5595. doi: 10.7717/peerj.5595 (PMC6173166; doi:10.7717/peerj.5595)
Supplement: Figure S1 [file peerj-06-5595-s012.pdf]

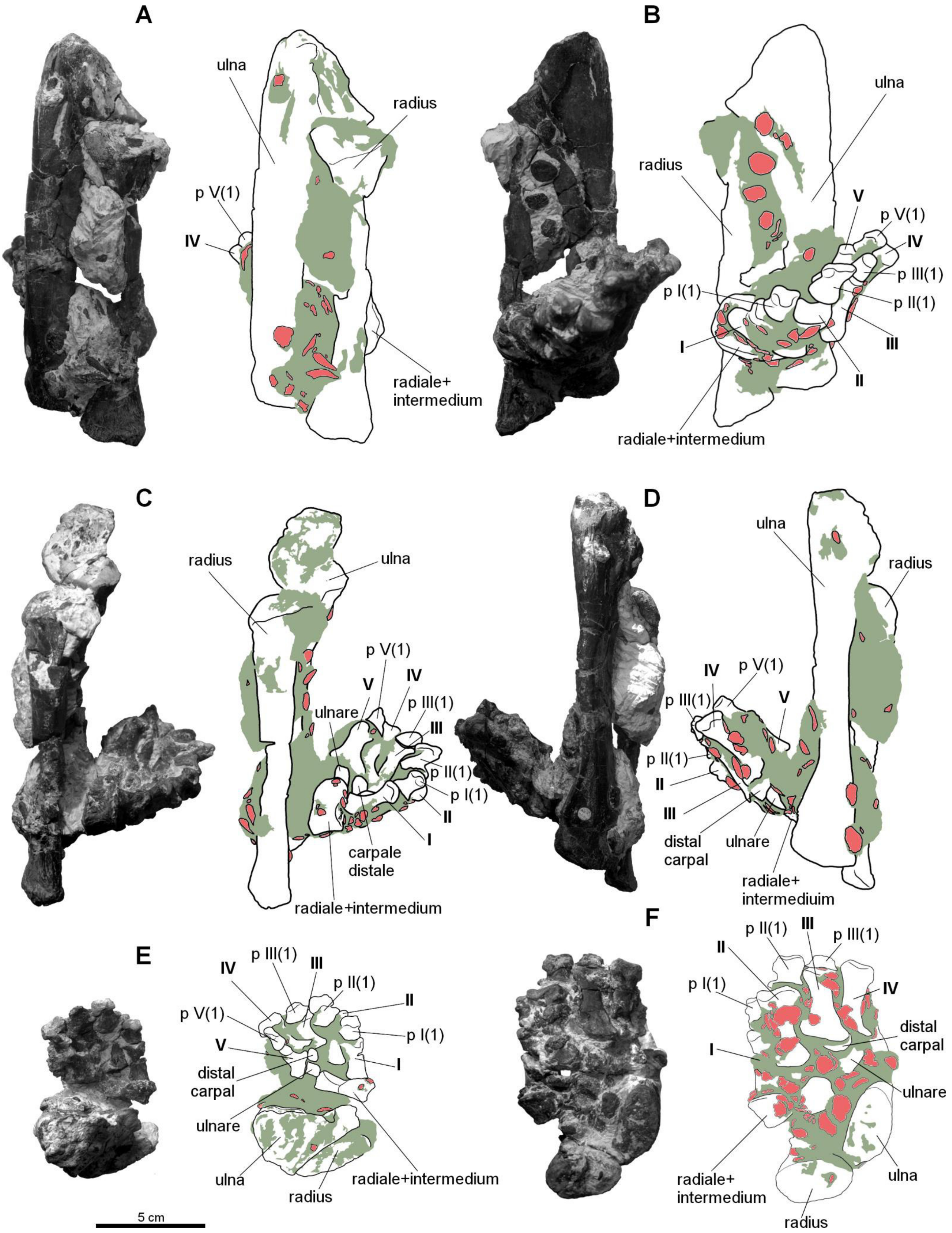

Supplementary Figure 1. Right manus and the forearm of the aetosaur *Stagonolepis olenkae*, Sulej 2010, ZPAL AbIII/2407. Photograph and schematic drawing of the specimen with the forearm in dorsal **(A)**, ventral **(B)**, medial **(C)**, and lateral view **(D)**. Photograph and schematic drawing of the specimen with hand bones exposed in ventral **(E)** and dorsal view **(F)**. The scale bar in E and F match to measurements of the forearm bones. Visible differences are an effect of foreshortening. In the schematic drawings osteoderms are marked red and the sediment is marked grey. All photographs and drawings are in the same scale.
