# Supplementary material for: Osteology of a forelimb of an aetosaur Stagonolepis olenkae (Archosauria: Pseudosuchia: Aetosauria) from the Krasiejów locality in Poland and its probable adaptations for a scratch-digging behavior
Source: PeerJ. 2018 Oct 2;6:e5595. doi: 10.7717/peerj.5595 (PMC6173166; doi:10.7717/peerj.5595)
Supplement: Figure S2 [file peerj-06-5595-s013.pdf]

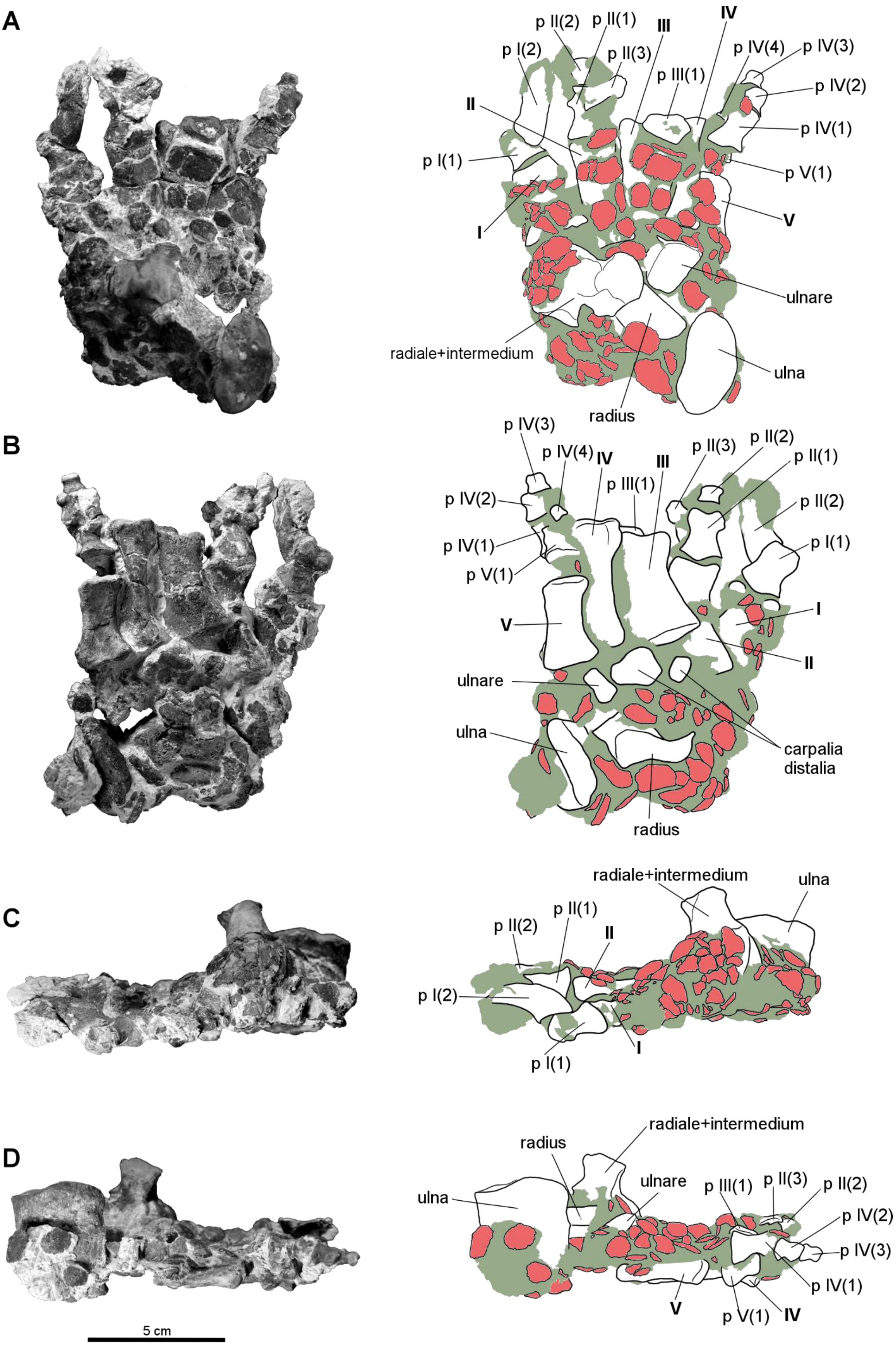

Supplementary Figure 2. Right manus and part of the forearm of the aetosaur *Stagonolepis olenkae*, Sulej 2010, ZPAL AbIII/3349/1. Photograph and schematic drawing of the specimen with the metacarpals and phalanges exposed in dorsal **(A)**, ventral **(B)**, medial **(C)** and lateral view **(D)**. In the schematic drawings osteoderms are marked red and the sediment is marked grey. All photographs and drawings are in the same scale.
