# Supplementary material for: Osteology of a forelimb of an aetosaur Stagonolepis olenkae (Archosauria: Pseudosuchia: Aetosauria) from the Krasiejów locality in Poland and its probable adaptations for a scratch-digging behavior
Source: PeerJ. 2018 Oct 2;6:e5595. doi: 10.7717/peerj.5595 (PMC6173166; doi:10.7717/peerj.5595)
Supplement: Figure S4 [file peerj-06-5595-s015.pdf]

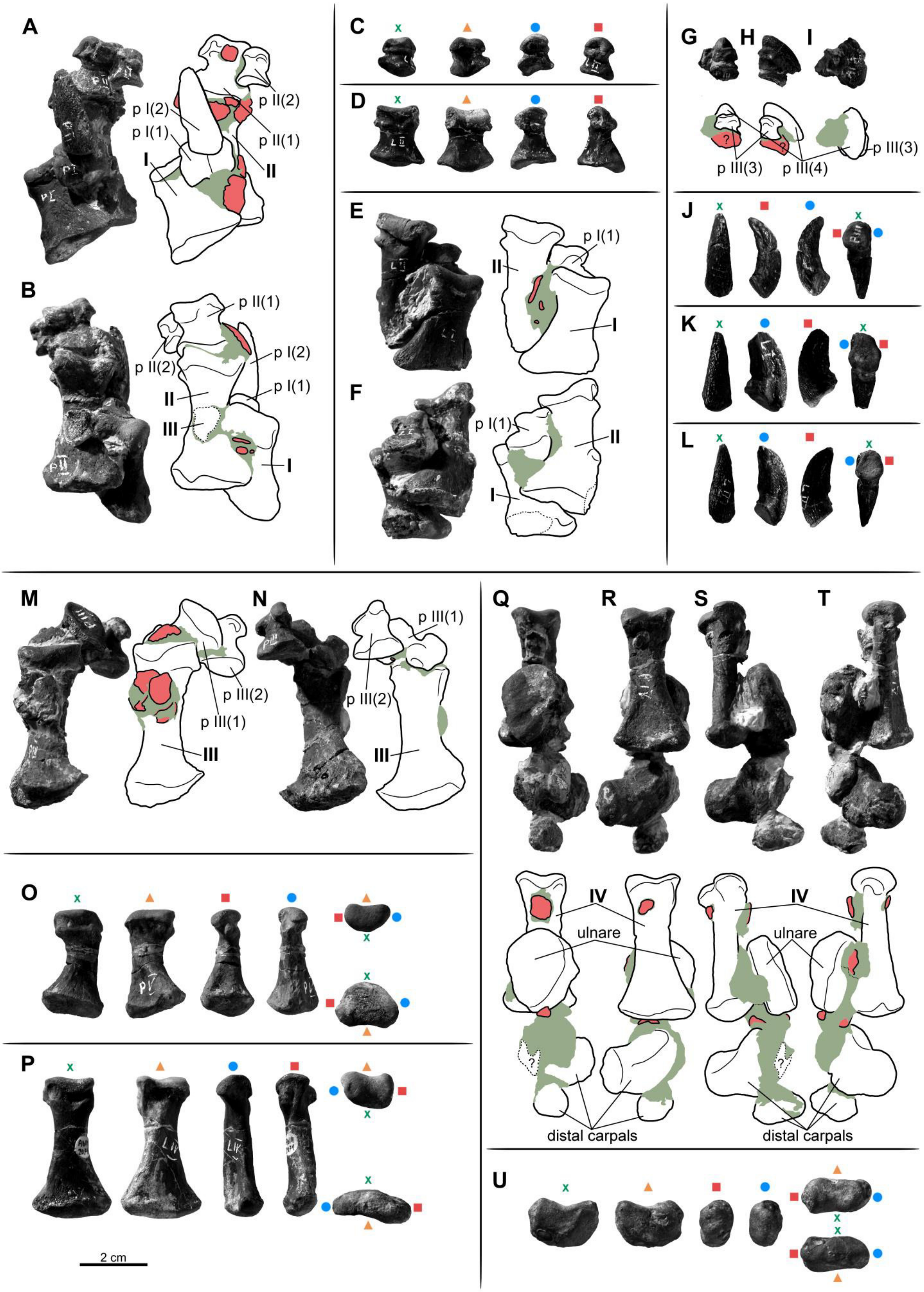

A
p I(2)
p I(1)
I
p II(2)
p II(1)
II
B
p II(1)
p II(2)
p I(2)
p I(1)
II
III
I
C
D
E
p I(1)
II
I
F
p I(1)
II
I
G
H
I
p III(3)
p III(4)
p III(3)
J
K
L
M
p III(2)
p III(1)
III
N
p III(1)
p III(2)
III
O
P
Q
R
S
T
IV
ulnare
distal carpals
IV
ulnare
distal carpals
U
2 cm

Supplementary Figure 4. Elements of the right and the left manus of the aetosaur *Stagonolepis olenkae*, Sulej 2010, ZPAL AbIII/2071. Schematic drawings and/or photographs **(A)** of metacarpals and phalanges of the first and second digit of the right manus in dorsal **(B)** and ventral view; **(C)** of the second phalanx of the second digit of the left manus; **(D)** of the first phalanx of the second digit of the left manus; **(E)** of metacarpals and phalanx of the first and second digit of the left manus in dorsal **(F)** and ventral view; **(G)** of the third phalanx and ungual of the third digit of the right manus, with the phalanx in dorsal, **(H)** lateral **(I)** and medial view; **(J)** of the ungual of the second digit of the right manus; **(K)** of the ungual of the first digit of the left manus; **(L)** of the ungual of the second digit of the left manus; **(M)** of the metacarpal and phalanges of the third digit of the right manus in dorsal **(N)** and ventral view; **(O)** of the of the metacarpal of the fifth digit of the right manus; **(P)** of the of the metacarpal of the fourth digit of the left manus; **(Q)** of the metacarpal of the fourth digit and carpals of the right manus, with the metacarpal in dorsal, **(R)** ventral **(S)** medial **(T)** and lateral view; **(U)** lunar-shaped carpal bone of the left manus. Symbols attached to some pictures show which surface is exposed in the photograph, with **(X)** for the dorsal, **(▲)** for the ventral, **(■)** for the medial, and **(●)** for the lateral, and how the surfaces are oriented in proximal and distal view. In (U) the symbols were added for technical reason and not necessarily reflect the real orientation of the bone. All photographs and drawings are in the same scale.
