# Supplementary material for: Osteology of a forelimb of an aetosaur Stagonolepis olenkae (Archosauria: Pseudosuchia: Aetosauria) from the Krasiejów locality in Poland and its probable adaptations for a scratch-digging behavior
Source: PeerJ. 2018 Oct 2;6:e5595. doi: 10.7717/peerj.5595 (PMC6173166; doi:10.7717/peerj.5595)
Supplement: Figure S6 [file peerj-06-5595-s017.pdf]

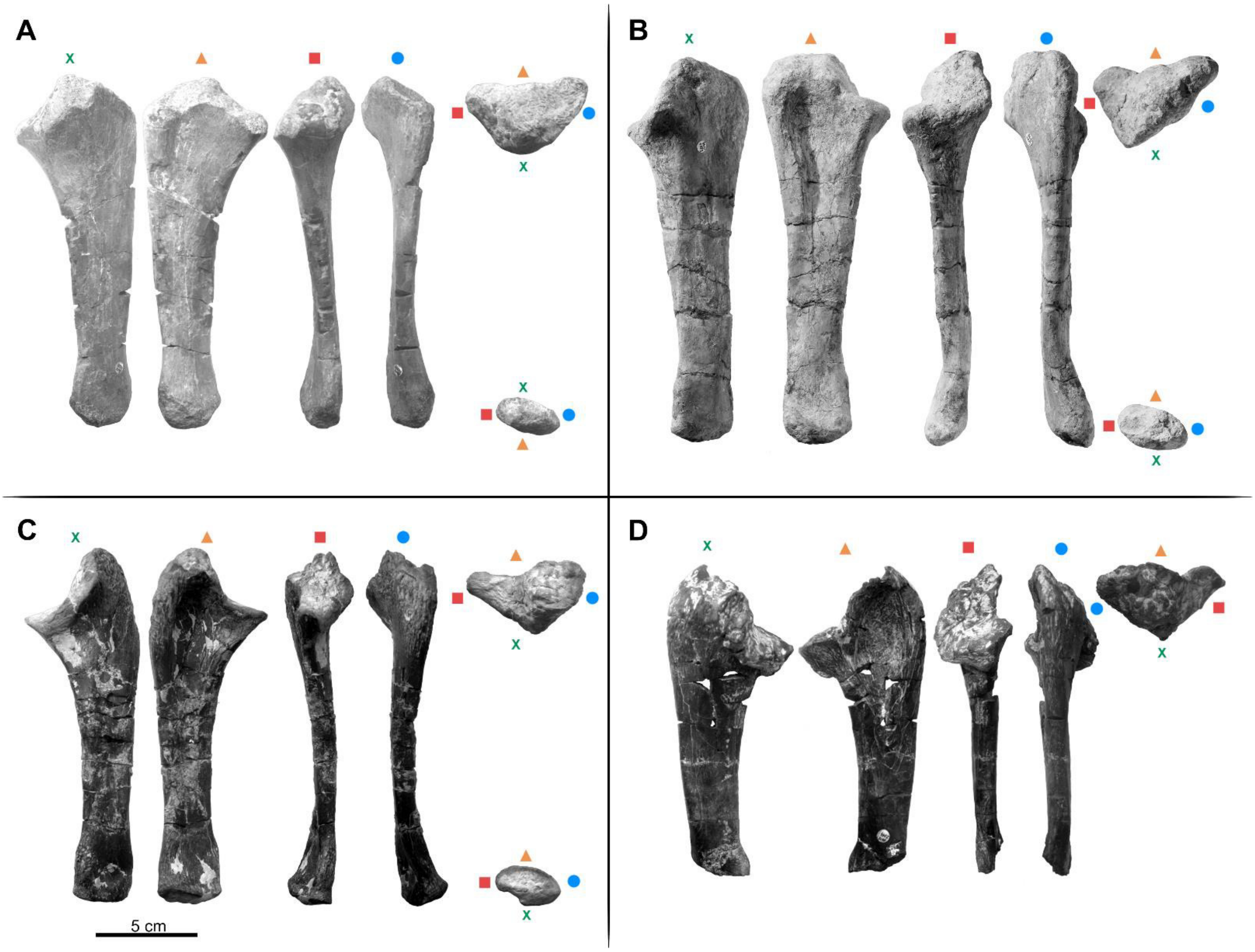

Supplementary Figure 6. Isolated ulnae of the aetosaur *Stagonolepis olenkae*, Sulej 2010. Photographs of **(A)** the left ulna ZPAL AbIII/1100/1; **(B)** the left ulna ZPAL AbIII/1179. **(C)** the left ulna ZPAL AbIII/3351. **(D)** the right ulna ZPAL AbIII/2014. Symbols attached to pictures show which surface is exposed in the photograph, with **(X)** for the dorsal, **(▲)** for the ventral, **(■)** for the medial, and **(●)** for the lateral, and how the surfaces are oriented in proximal and distal view. All photographs are in the same scale.
