# Supplementary material for: Osteology of a forelimb of an aetosaur Stagonolepis olenkae (Archosauria: Pseudosuchia: Aetosauria) from the Krasiejów locality in Poland and its probable adaptations for a scratch-digging behavior
Source: PeerJ. 2018 Oct 2;6:e5595. doi: 10.7717/peerj.5595 (PMC6173166; doi:10.7717/peerj.5595)
Supplement: Figure S8 [file peerj-06-5595-s019.pdf]

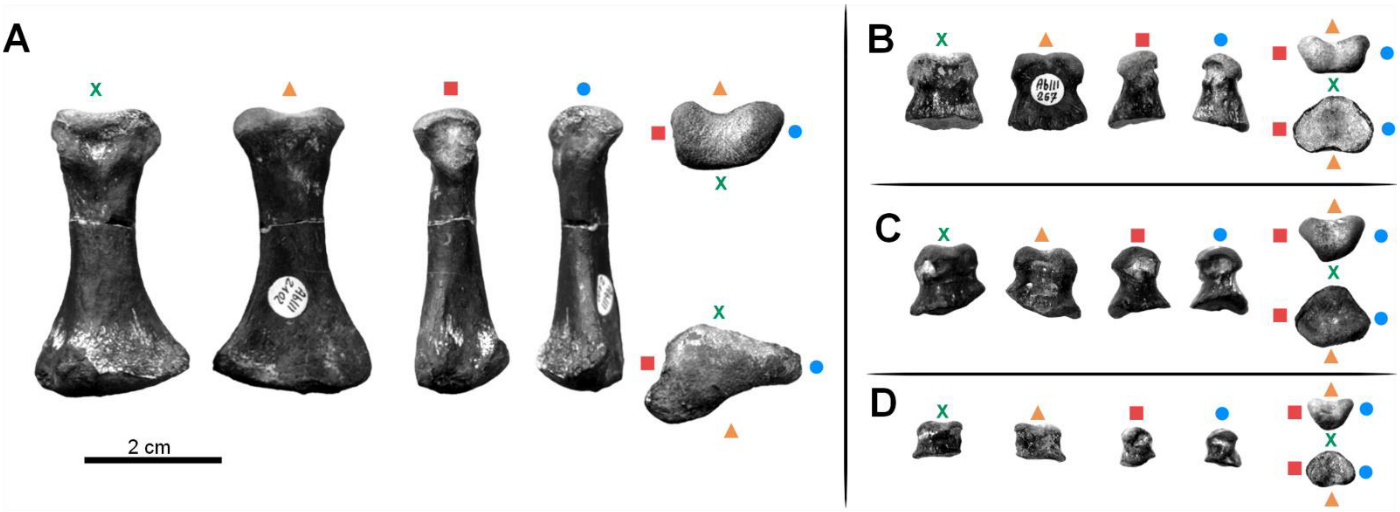

Supplementary Figure 8. Isolated elements of manus of the aetosaur *Stagonolepis olenkae*, Sulej 2010. **(A)** the right metacarpal of the digit IV, ZPAL AbIII/2102; **(B)** the second phalanx of the digit III of a left manus, ZPAL AbIII/267. **(C)** the second phalanx of the digit II of a right manus, ZPAL AbIII/3352. **(D)** the third phalanx of the digit III of a left manus, ZPAL AbIII/3353. Symbols attached to pictures show which surface is exposed in the photograph, with **(X)** for the dorsal, **(▲)** for the ventral, **(■)** for the medial, and **(●)** for the lateral, and how the surfaces are oriented in proximal and distal view. All photographs are in the same scale.
