# Supplementary material for: Osteology of a forelimb of an aetosaur Stagonolepis olenkae (Archosauria: Pseudosuchia: Aetosauria) from the Krasiejów locality in Poland and its probable adaptations for a scratch-digging behavior
Source: PeerJ. 2018 Oct 2;6:e5595. doi: 10.7717/peerj.5595 (PMC6173166; doi:10.7717/peerj.5595)
Supplement: Figure S9 [file peerj-06-5595-s020.pdf]

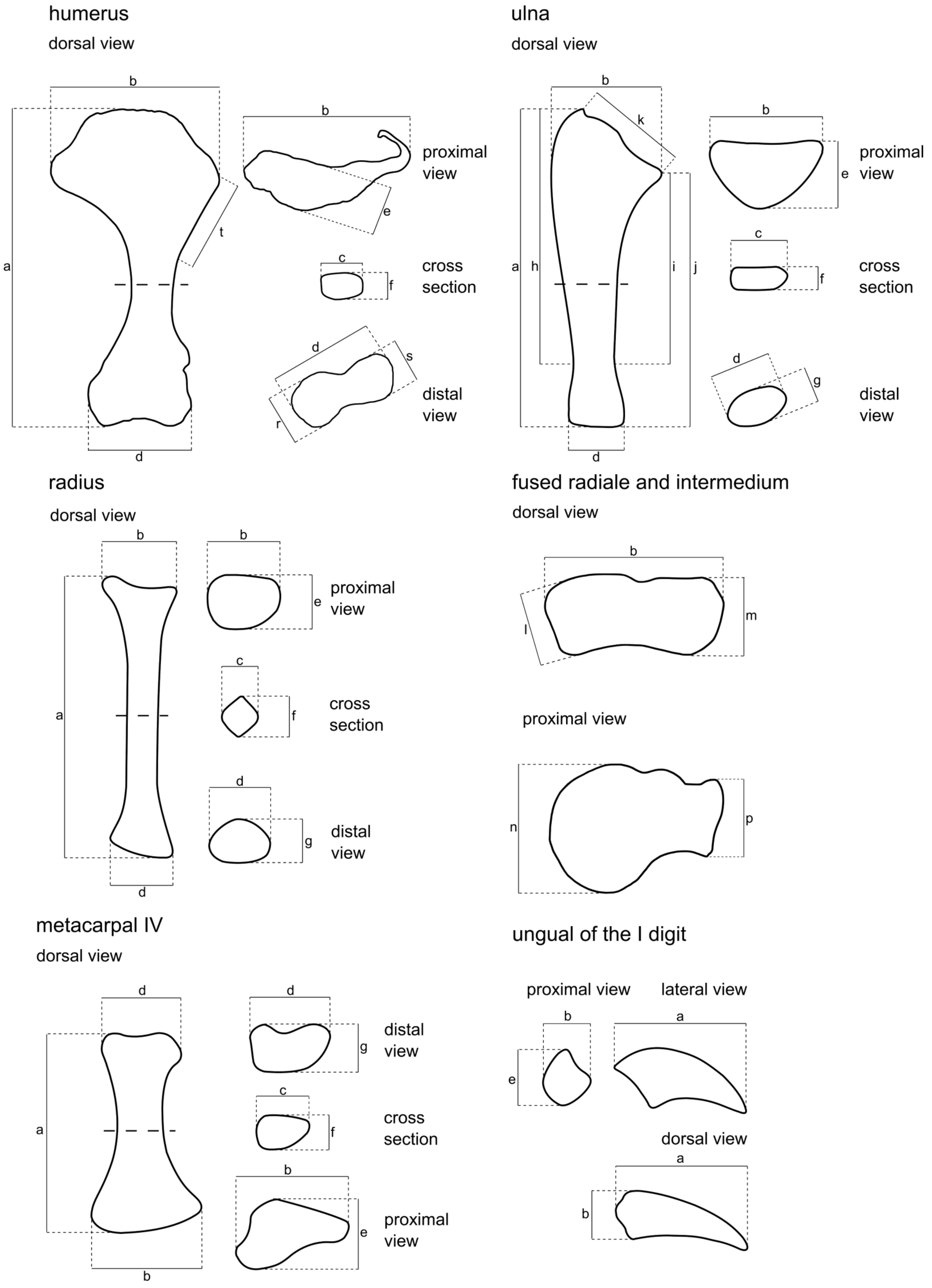

Supplementary Figure 9. Schematic drawings of the forelimbs bones of the *Stagonsolepis olenkae* from Krasiejów locality in Poland, showing how the measurements of the bones were taken.
