# Supplementary material for: Osteology of a forelimb of an aetosaur Stagonolepis olenkae (Archosauria: Pseudosuchia: Aetosauria) from the Krasiejów locality in Poland and its probable adaptations for a scratch-digging behavior
Source: PeerJ. 2018 Oct 2;6:e5595. doi: 10.7717/peerj.5595 (PMC6173166; doi:10.7717/peerj.5595)
Supplement: Figure S10 [file peerj-06-5595-s021.pdf]

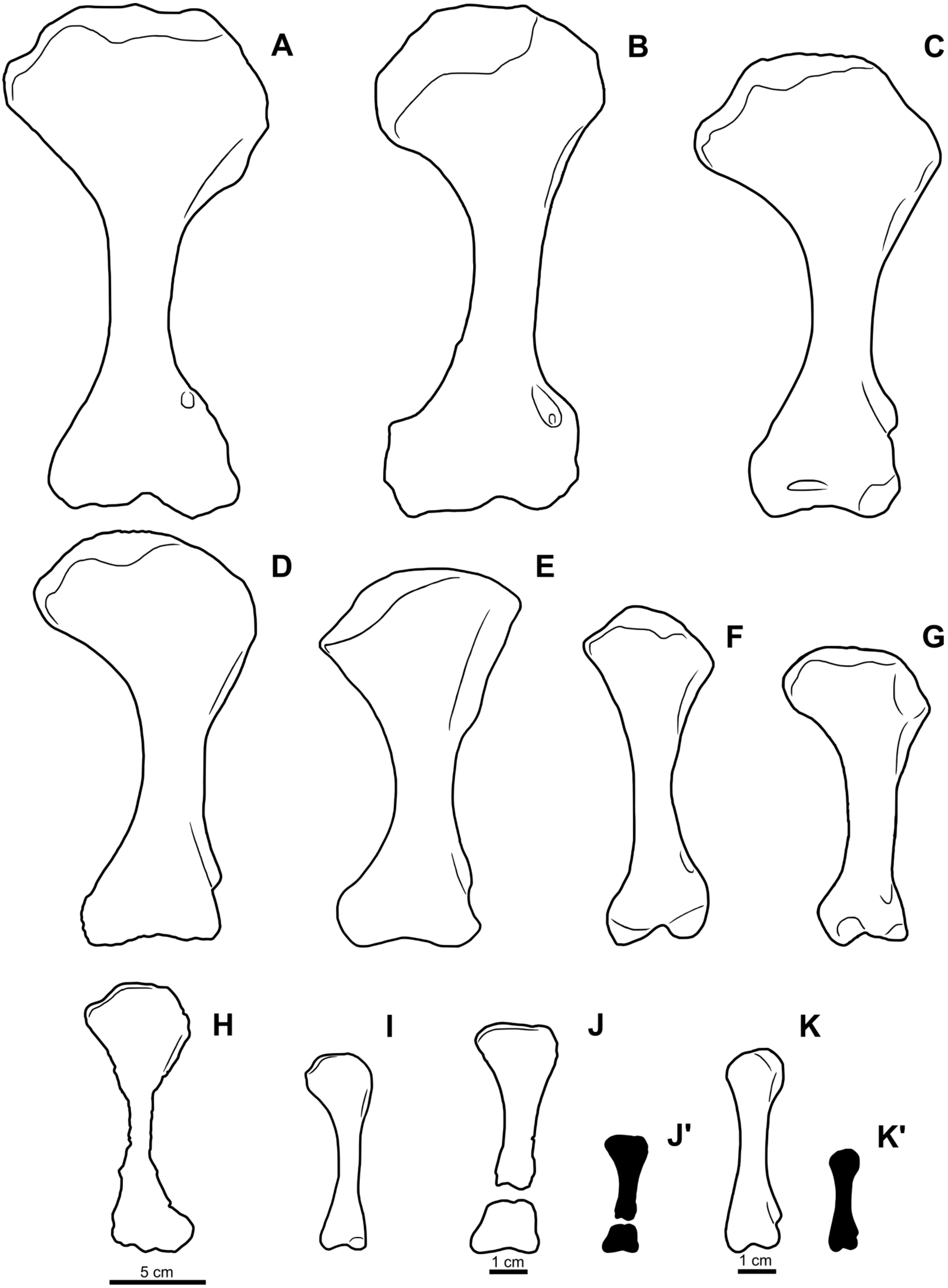

A
B
C
D
E
F
G
H
I
J
J'
K
K'
5 cm
1 cm
1 cm

Supplementary Figure 10. Schematic drawings of the humeri in different aetosaur species. **(A)** *Desmatosuchus spurensis*, Case 1920, sensu Parker 2008 (based on the photograph of spec. UCMP A269/32168, fig. 90b, in Long and Murry 1995). **(B)** *Longosuchus meadei*, Sawin 1947, redescribed as new genus by Hunt and Lucas 1990 (based on the drawing, fig. 4a, in Sawin 1947). **(C)** *Stagonolepis olenkae*, Sulej 2010 (based on the spec. ZPAL AbIII/1175) **(D)** *Stagonolepis robertsoni,* Agassiz 1844 (based on the drawing, fig. 14a, in Walker 1961). **(E)** "*Argentinosuchus bonapartei*" (based on the photograph of spec. PVL 2091, fig. 5-2, in Heckert and Lucas 2002). **(F)** *Typothorax coccinarum*, Cope 1875 (based on the drawing of spec. UCMP V2816 34240 70/J9, fig. 4.10b, in Martz 2002). **(G)** *Neoaetosauroides engaeus*, Bonaparte 1969 (based on the drawing of spec. PVL. 3525, fig. 38a, in Bonaparte 1971). **(H)** *Aetobarbakinoides brasiliensis*, Desojo *et al.* 2012 (based on the photograph, fig. 12b, in Desojo *et al.* 2012). **(I)** *Aetosauroides scagliai,* Casamiquela 1960 (based on the photograph of spec. PVL 2073, fig. 3-2, in Heckert and Lucas 2002). **(J,J')** *Polesinesuchus aureolii*, Roberto-da-Silva *et al.* 2014 (based on the photograph of spec. ULBRAPVT003, fig. 19b, in Roberto-da-Silva *et al.* 2014). **(K,K')** *Aetosaurus ferratus*, Frass 1877, (based on the drawing of spec. SMNS 5770 (S- 5), fig. 10f, in Schoch 2007). All pictures present humeri in dorsal view. All drawings, except of J and K, are in the same scale.
