# Supplementary material for: Osteology of a forelimb of an aetosaur Stagonolepis olenkae (Archosauria: Pseudosuchia: Aetosauria) from the Krasiejów locality in Poland and its probable adaptations for a scratch-digging behavior
Source: PeerJ. 2018 Oct 2;6:e5595. doi: 10.7717/peerj.5595 (PMC6173166; doi:10.7717/peerj.5595)
Supplement: Figure S11 [file peerj-06-5595-s022.pdf]

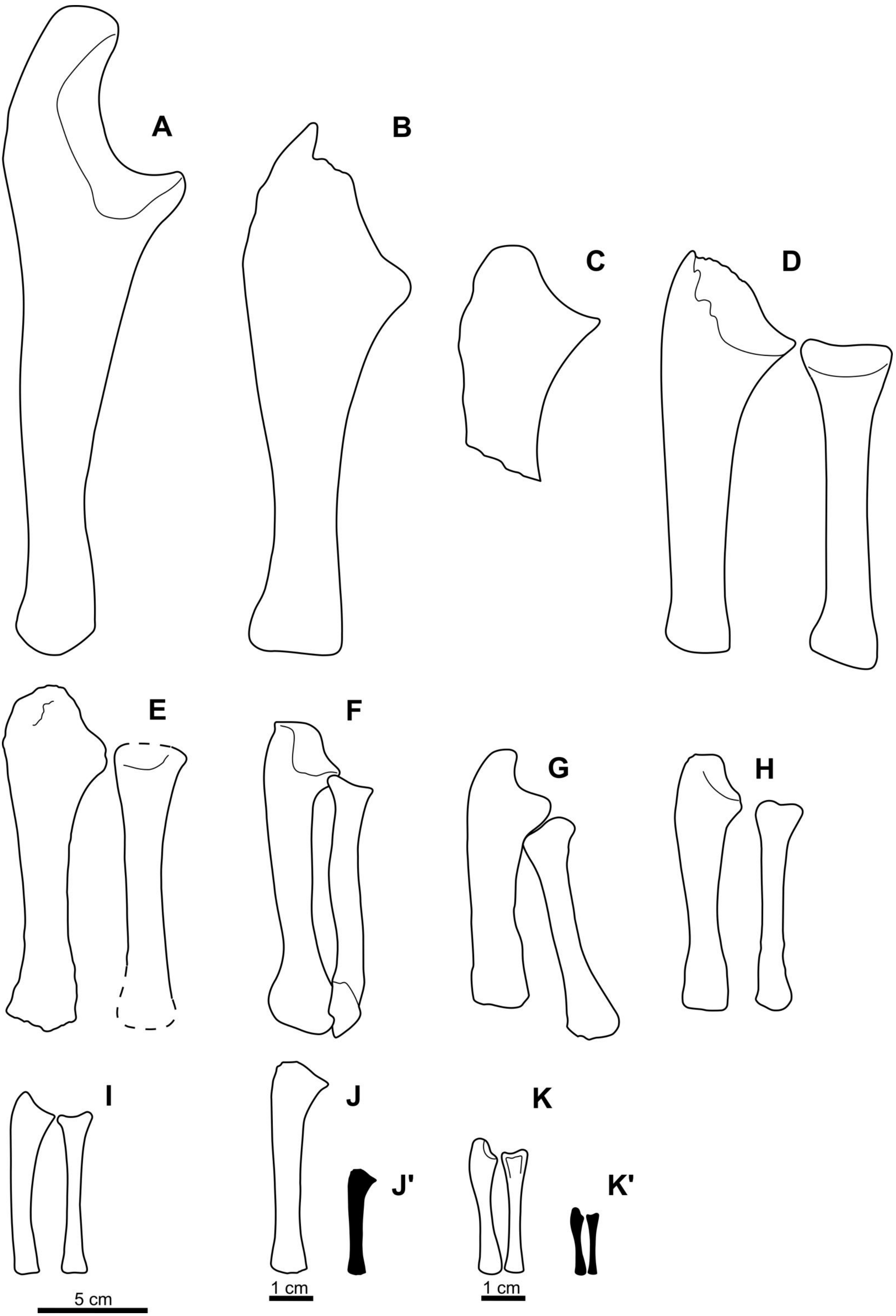

A
B
C
D
E
F
G
H
I
J
J'
K
K'
5 cm
1 cm
1 cm

Supplementary Figure 11. Schematic drawings of the forearm bones in different aetosaur species. **(A)** Ulna of *Desmatosuchus smalli*, Parker 2005 (based on the drawing of spec. TTUP 9170, fig. 8c, in Small 1985). **(B)** Ulna of *Longosuchus meadei*, Sawin 1947, redescribed as new genus by Hunt and Lucas 1990 (based on the drawing, fig. 4c, in Sawin 1947). **(C)** Ulna of "*Argentinosuchus bonapartei*" (based on the photograph of spec. PVL 2091, fig. 5-4, in Heckert and Lucas 2002). **(D)** Ulna and radius of *Stagonolepis olenkae,* Sulej 2010 (based on the spec. ZPAL AbIII/2407). **(E)** Ulna and radius of *Stagonolepis robertsoni*, Agassiz 1844 (based on the draw-ing, fig. 14c, e, in Walker 1961). **(F)** Ulna and radius of *Neoaetosauroides engaeus*, Bonaparte1969 (based on the drawing of spec. PVL. 3525, fig. 38 b, in Bonaparte 1971). **(G)** Ulna and radius of *Typothorax coccinarum*, Cope 1875 (based on the photograph of spec. NMMNH P-56299, fig. 4b, in Heckert *et al.* 2010). **(H)** Ulna and radius of *Typothorax antiquum*, Lucas *et al.* 2002 (based on photographs of spec. NMMNH P 36075, fig. 7a, c, in Lucas *et al.* 2002). **(I)** Ulna and radius of *Aetosauroides scagliai,* Casamiquela 1960 (based on the photographof spec. PVL 2073, fig. 3-4, 6, in Heckert and Lucas 2002). **(J,J')** Ulna of *Polesinesuchus aureolii*, Roberto-da-Silva *et al.* 2014 (based on the photograph of spec. ULBRAPVT003, fig. 21a, in Roberto-da-Silva *et al.* 2014) **(K,K')** Ulna and radius of *Aetosaurus ferratus*, Frass 1877, (based on the drawing of spec. SMNS 5770 (S-10), fig. 10g, in Schoch 2007). All pictures present forearm bones in dorsal view (except of B in ventral view). All drawings are in the same scale, except of J and K.
