# Supplementary material for: Osteology of a forelimb of an aetosaur Stagonolepis olenkae (Archosauria: Pseudosuchia: Aetosauria) from the Krasiejów locality in Poland and its probable adaptations for a scratch-digging behavior
Source: PeerJ. 2018 Oct 2;6:e5595. doi: 10.7717/peerj.5595 (PMC6173166; doi:10.7717/peerj.5595)
Supplement: Figure S12 [file peerj-06-5595-s023.pdf]

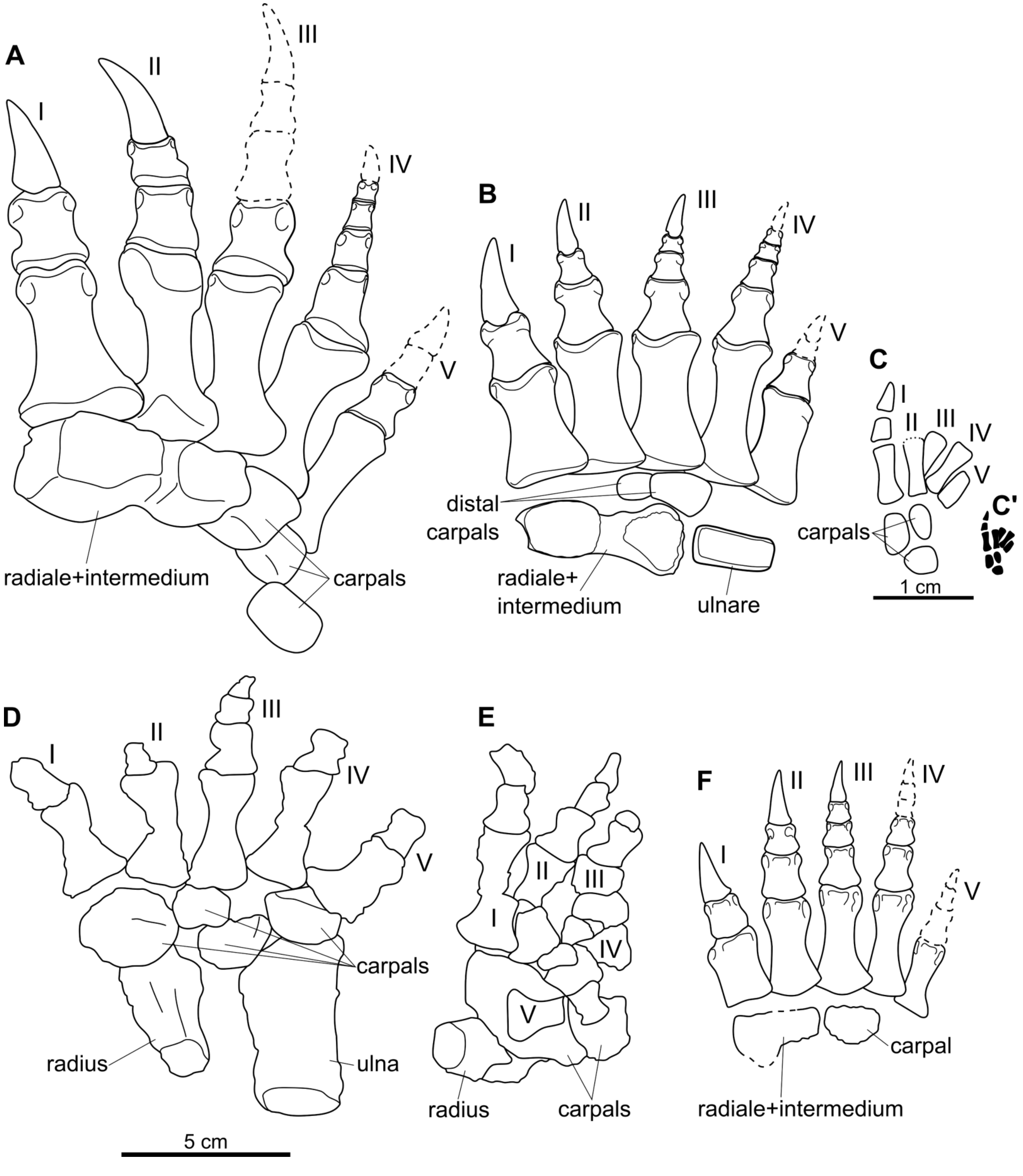

Supplementary Figure 12. Schematic drawings of the manus bones in different aetosaur species. **(A)** *Longosuchus meadei*, Sawin 1947, redescribed as new genus by Hunt and Lucas 1990 (based on the drawing, fig. 3c, in Sawin 1947). **(B)** *Stagonolepis olenkae*, Sulej 2010 (size based on the spec. ZPAL AbIII/3349/1). **(C, C')** *Aetosaurus ferratus*, Frass 1877 (based on the drawing of spec. SMNS 5770 (S-10), fig. 10g, in Schoch 2007). **(D)** *Typothorax coccinarum*, Cope 1875 (based on the photograph of spec. MCZ 1487, fig. 4a, in Lucas and Heckert 2011). **(E)** *Typothorax coccinarum*, Cope 1875, (based on the photograph of spec. MCZ 1488, fig. 4c, in Lucas and Heckert 2011). **(F)** *Stagonolepis robertsoni*, Agassiz 1844 (based on the drawing, fig. 14g, in Walker 1961). All pictures, present autopodium bones in dorsal view (except of D and E in ventral view). All drawings, except of C, are in the same scale.
